# Boron clusters as broadband membrane carriers

**DOI:** 10.1038/s41586-022-04413-w

**Published:** 2022-03-23

**Authors:** Andrea Barba-Bon, Giulia Salluce, Irene Lostalé-Seijo, Khaleel. I. Assaf, Andreas Hennig, Javier Montenegro, Werner M. Nau

**Affiliations:** 1 Department of Life Sciences and Chemistry, Jacobs University Bremen, Bremen, Germany; 2 Departamento de Química Orgánica e Centro Singular de Investigación en Química Biolóxica e Materiais Moleculares (CIQUS), Universidade de Santiago de Compostela, Santiago de Compostela, Spain

**Keywords:** Drug delivery, Molecular capsules

## Abstract

The membrane translocation of hydrophilic substances constitutes a challenge for their application as therapeutic compounds and labelling probes^1–4^. To remedy this, charged amphiphilic molecules have been classically used as carriers^3,5^. However, such amphiphilic carriers may cause aggregation and non-specific membrane lysis^6,7^. Here we show that globular dodecaborate clusters, and prominently B_12_Br_12_^2−^, can function as anionic inorganic membrane carriers for a broad range of hydrophilic cargo molecules (with molecular mass of 146–4,500 Da). We show that cationic and neutral peptides, amino acids, neurotransmitters, vitamins, antibiotics and drugs can be carried across liposomal membranes. Mechanistic transport studies reveal that the carrier activity is related to the superchaotropic nature of these cluster anions^8–12^. We demonstrate that B_12_Br_12_^2−^ affects cytosolic uptake of different small bioactive molecules, including the antineoplastic monomethyl auristatin F, the proteolysis targeting chimera dBET1 and the phalloidin toxin, which has been successfully delivered in living cells for cytoskeleton labelling. We anticipate the broad and distinct delivery spectrum of our superchaotropic carriers to be the starting point of conceptually distinct cell-biological, neurobiological, physiological and pharmaceutical studies.

## Abstract (web summary)

The superchaotropic nature of globular boron cluster anions enables direct passage of a wide range of molecules across lipid membranes.

## Main

**Fig. 1:**
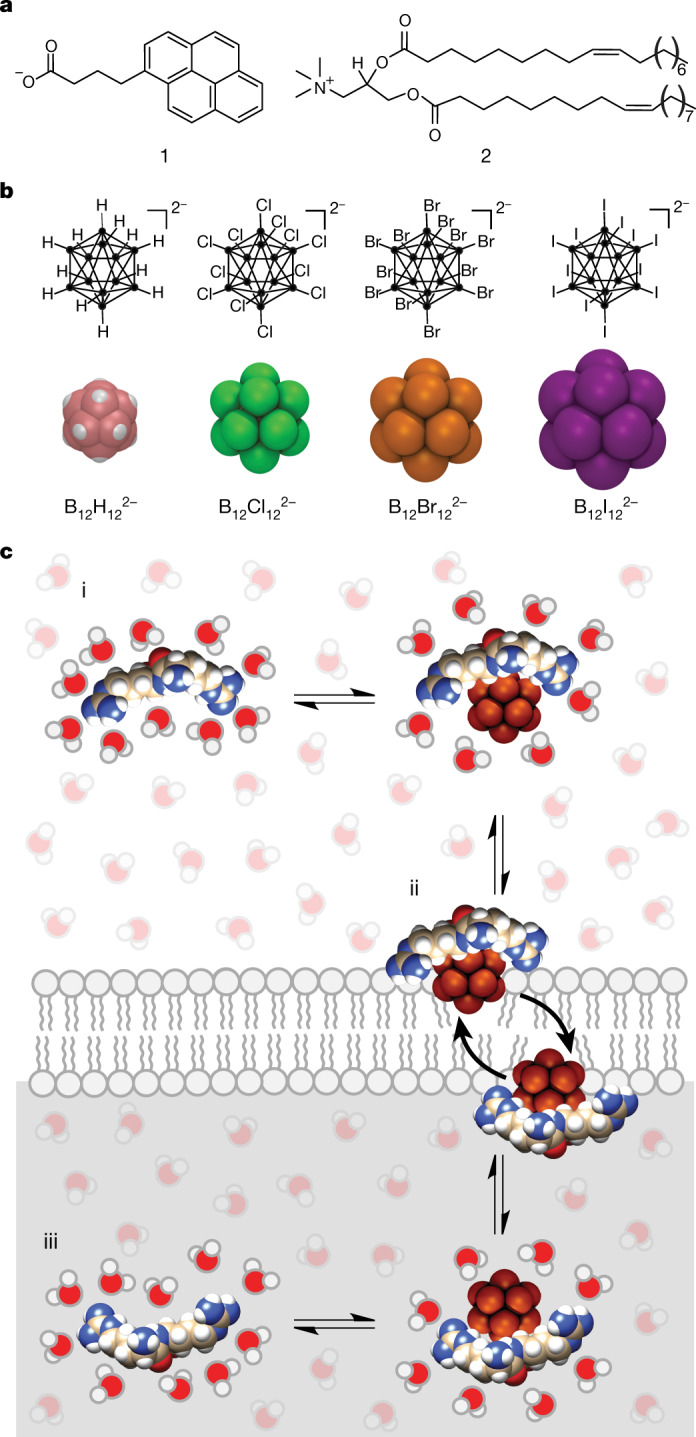
Classical amphiphilic carriers/activators and boron clusters. **a**, Established amphiphilic compounds that act as counterion activator (**1**) or as carrier (**2**). **b**, Chemical structures (top, ● represents boron) and space-filling molecular models (bottom) of dodecaborate (B_12_X_12_^2−^) clusters with increasing diameter (8.0–11.8 Å, from refs. ^8,9^). **c**, Direct membrane and cargo translocation by superchaotropic clusters. (i) Hydrophilic molecules (for example, an R_2_ peptide) present a high barrier against desolvation and, thus, cannot interact with/cross the lipid membrane. (ii) The (enthalpy-driven) chaotropic interactions drive desolvation of the cargo and facilitate cargo membrane partitioning and direct translocation. (iii) After membrane passage, the reversible nature of the chaotropic interaction leads to dissociation of the complex and release of the cargo.

The design of carriers that affect non-lytic membrane passage of bioactive cargos presents a critical challenge in chemistry and materials science^1,2^. To surmount the membrane barrier, artificial transporters have been developed, including synthetic pores^3^, ionophores^4,13^, macrocycles^14,15^, lipids^16^, nanoparticles^17^, counterion activators^18,19^, cationic penetrating peptides^20,21^ and liposomes^22^. In this search for synthetic membrane carriers, one conceptual approach has prevailed so far. It entails the design of amphiphilic molecules, combining ionic head groups with hydrophobic tails^2^, in which the latter serve as anchors to ensure membrane affinity and the former electrostatically attract the cargo to trigger membrane transport of the resulting charge-neutralized complexes^23^. The prime exponents of these carriers are exemplified by anionic amphiphiles (**1**)^18^ or cationic lipids (**2**)^22^ (Fig. 1a). The latter (**2**) presents a typical example of an amphiphilic nanocarrier and the former, pyrenebutyrate (**1**), is the prototype of a counterion activator, which activates the transport of cationic hydrophilic peptides by the transient enhancement of their amphiphilicity^5,24^. Despite rapid progress in the field, the development of new amphiphilic membrane transporters meets with intrinsic limitations due to their aggregation tendency^6,19,25^, unspecific binding^15,21^, endocytic entrapment^6,25–28^, membrane-lytic propensity^27^ and toxicity^15,27^.

We now introduce globular boron cluster anions (Fig. 1b) as an orthogonal class of direct membrane carriers that abandon the classical amphiphilic topology. Boron clusters are inorganic rather than organic species, and they draw their membrane affinity from being superchaotropic^8^ rather than amphiphilic. As a consequence, and in contrast to conventional membrane transporters, boron clusters are highly water soluble and do not encapsulate, nor form aggregates, with their cargo. Besides these fundamental chemical and physical differences, we find that superchaotropic clusters also affect direct membrane transport of a broad range of hydrophilic cargos.

The globular boron clusters of interest are stable anions with a permanent double-negative charge, weak ligating properties and high biocompatibility^29^. They have found earlier applications, amongst others, in boron neutron capture cancer therapy^30^. Recently, we identified dodecaborate clusters of the type B_12_X_12_^2−^ (X = H, Cl, Br and I) as being superchaotropic in nature, that is, their chaotropic properties exceed those of the most chaotropic anions on the Hofmeister scale (ClO_4_^−^, SCN^−^ and PF_6_^−^)^9^. Therefore, on a continuous scale for aqueous solvation, their properties start to resemble those of ionic hydrophobic species, despite large structural and physicochemical differences, with the result that they begin to display a generic propensity to dynamically associate to hydrophobic areas^8^, including lipid bilayers^8,31,32^. These recently revealed similarities^10–12,33^ led us to hypothesize that boron clusters, and potentially other large ions with superchaotropic character, could activate the direct membrane transport of hydrophilic molecular cargos.

## Carrier activity in model membranes

**Fig. 2:**
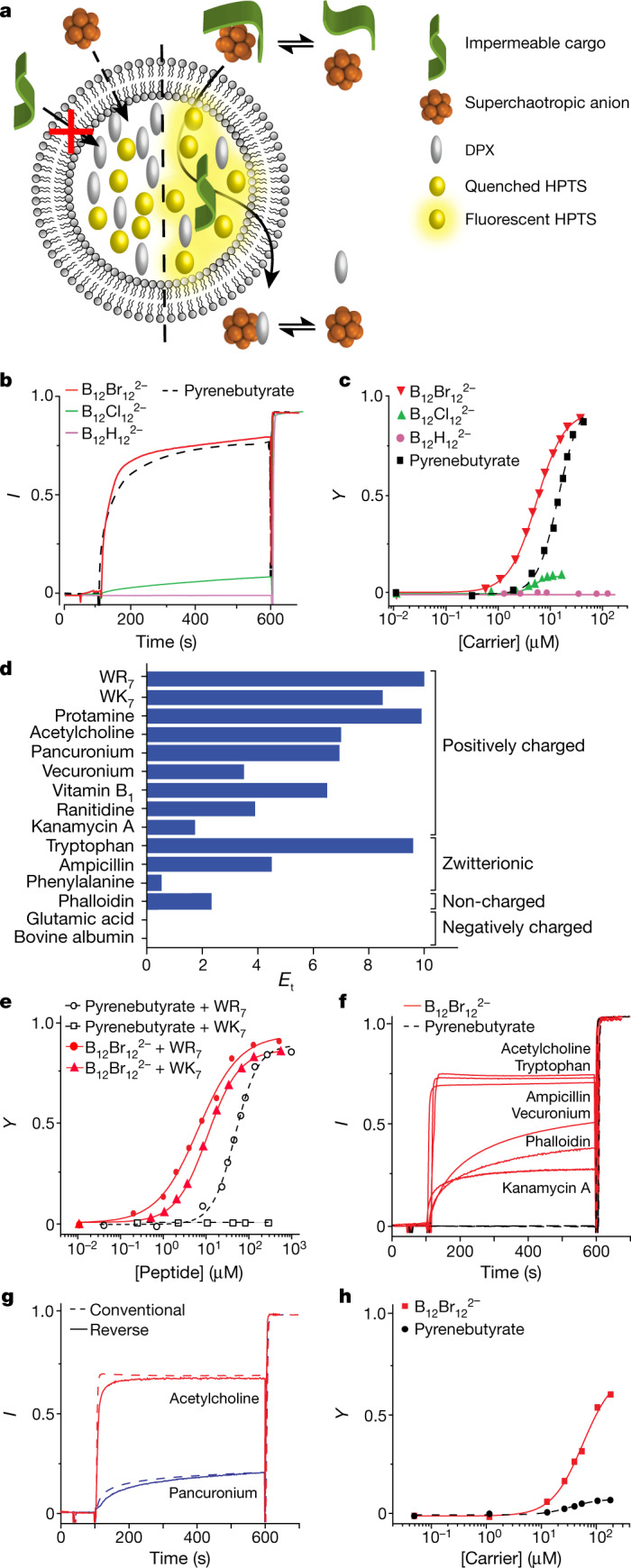
Carrier activity obtained from the HPTS/DPX assay. **a**, Schematic representation of the transport of otherwise impermeable analytes facilitated by superchaotropic cluster anions with encapsulated HPTS/DPX probe/quencher pair used for signalling. In the presence of suitable carriers, cargo is carried into the vesicles and the cationic quencher DPX is transported from the endoliposomal phase to the bulk, resulting in increased fluorescence of HPTS as the optical signal of successful cargo transport. **b**, Changes in HPTS emission (*I*) (*λ*_ex_ = 413 nm, *λ*_em_ = 511 nm) in EYPC⊃HPTS/DPX vesicles as a function of time during the addition of the membrane carriers B_12_Br_12_^2−^ and B_12_Cl_12_^2−^, the established activator pyrenebutyrate (**1**), as well as the inactive parent B_12_H_12_^2−^, as negative control; clusters (150 µM) added at *t* = 50 s, WR_7_ at *t* = 100 s and TX-100 at *t* = 600 s, for calibration. **c**, Dependence of fractional activity *Y* for WR_7_ transport on the concentration of different clusters, and the corresponding Hill curve fits in comparison to reference compound﻿ **1**. **d**, Transport efficiency of B_12_Br_12_^2−^ towards selected impermeable analytes of biological/medicinal relevance; values obtained by non-linear regression analysis (Hill equation), see Extended Data Table 1. **e**, Comparable transport of WR_7_ (circles) and WK_7_ (triangles﻿ or squares) activated by B_12_Br_12_^2−^ (red, solid lines) versus exclusive transport of WR_7_ activated by **1** (black, dashed lines). **f**, Successful transport of membrane-impermeable analytes (100 µM) affected by B_12_Br_12_^2−^ (red, solid line) in the HPTS/DPX assay and transport failure for the established amphiphilic activator pyrenebutyrate (**1**, black, dashed lines). **g**, ‘Conventional’ (dashed line) versus reverse addition (solid line), demonstrating that the sequence of addition does not markedly affect transport efficiency. **h**, Transport efficiency in anionic DMPE/DPPG/CHOL⊃HPTS/DPX vesicles as a function of B_12_Br_12_^2−^ (red, solid line) versus pyrenebutyrate (**1**, black, dashed line) concentration at constant cargo concentration (WR_7_). Concentrations of carrier and cargo, when fixed, were 40 and 20 µM, respectively, unless explicitly stated. Source data

Prototypical cationic peptides, such as oligoarginines, do not translocate through zwitterionic phosphocholine lipid vesicles on their own^5,19,34^. Therefore, the capability of boron clusters to activate the transport of a hepta-arginine peptide (WR_7_) was investigated first in large unilamellar vesicles. The well-established HPTS/DPX assay^35^, which uses 8-hydroxypyrene-1,3,6-trisulfonate (HPTS) and *p*-xylene-bis-pyridinium (DPX), was implemented to monitor peptide-transport activation (Fig. 2a). In a typical time-resolved fluorescence experiment, HPTS emission was monitored during the sequential addition of the globular cluster carrier (time *t* = 50 s) and the peptide cargo (*t* = 100 s; Fig. 2b). A surfactant (Triton X-100) was added at the end of the experiment (*t* = 600 s) to release all vesicle content and normalize the fluorescence intensity data.

**Extended Data Fig. 1:**
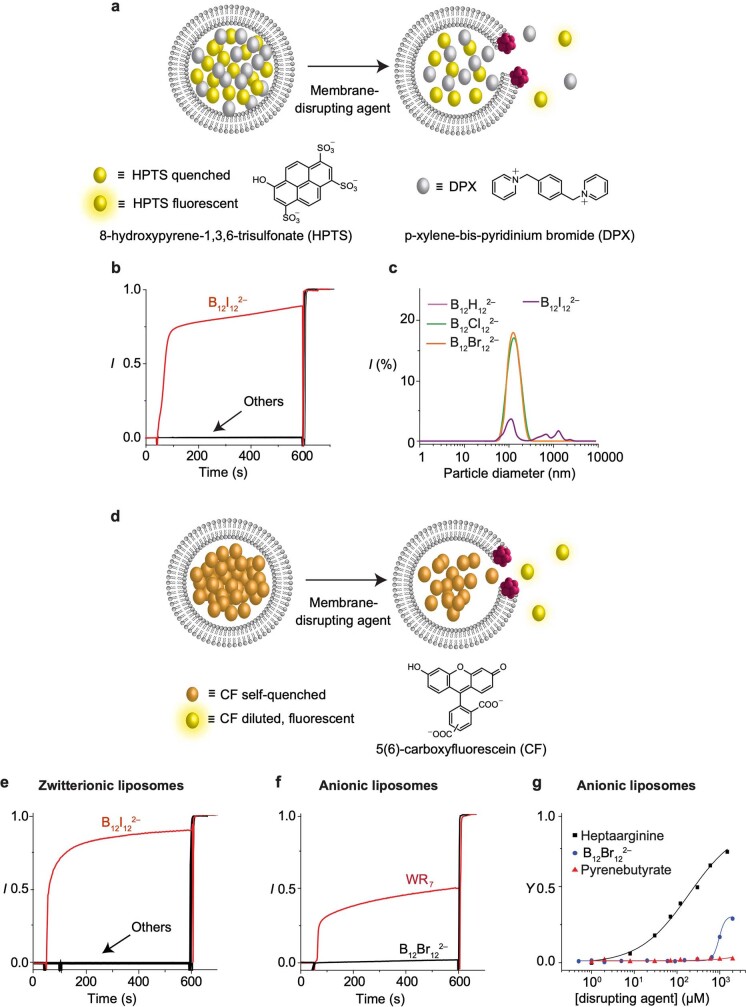
Assays testing for stability *vs* leakage of the vesicles. **a**, Response of the HPTS/DPX assay towards a membrane-disrupting agent and chemical structures of the fluorescent dye HPTS and the quencher DPX. In this assay, large unilamellar vesicles are loaded with the HPTS/DPX probe/quencher pair, such that the fluorescence of HPTS is quenched. Although the addition of a membrane-compatible carrier causes only a response in the presence of suitable cargo (see Fig. 2 in main text) the addition of a membrane-disrupting agent (B_12_I_12_^2−^) induces probe and quencher efflux, resulting in dilution with a concomitant sudden increase in fluorescence even in the absence of cargo. **b**, Changes in fractional HPTS emission intensity (*λ*_ex_ = 413 nm, *λ*_em_ = 511 nm) of EYPC⊃HPTS/DPX vesicles (13 µM phospholipids in 10 mM Tris, 107 mM NaCl, pH 7.4) during the addition of 40 µM of clusters. The cluster B_12_I_12_^2−^ (red trace) was identified as lytic agent because the mere addition of the cluster led to a fluorescence increase. None of the other clusters (B_12_H_12_^2−^, B_12_Cl_12_^2−^, and B_12_Br_12_^2−^) produced any perturbation in the vesicle membrane as monitored by a constant fluorescence intensity (baseline, black traces). **c**, Size distribution (by DLS) of EYPC⊃HPTS/DPX vesicles before and after addition of 200 µM cluster and 20 µM WR_7_ to differentiate the clusters that retain membrane integrity (B_12_H_12_^2−^, B_12_Cl_12_^2−^, and B_12_Br_12_^2−^) from the one that lyses the vesicles (B_12_I_12_^2−^). **d**, Working principle of the carboxyfluorescein (CF) leakage assay principle and chemical structure of the fluorescent probe. In this assay, large unilamellar vesicles are loaded with CF at high concentration, such that the fluorescence is quenched. The addition of a membrane-disrupting agent induces dye efflux, resulting in a CF dilution with the concomitant increase in fluorescence. **e**, Changes in fractional CF emission intensity (*λ*_ex_ = 492 nm, *λ*_em_ = 517 nm) of EYPC⊃CF vesicles (13 µM phospholipids in 10 mM HEPES, 107 mM NaCl, pH 7.5) during the addition of 40 µM of clusters and 20 µM WR_7_. The cluster B_12_I_12_^2−^ (red trace) was identified as lytic agent because its mere addition led to a fluorescence increase. None of the other clusters (B_12_H_12_^2−^, B_12_Cl_12_^2−^, and B_12_Br_12_^2−^) produced any perturbation in the vesicle membrane (even in the presence of WR_7_) as monitored by a constant fluorescence intensity (baseline). **f**, Changes in the fractional CF emission intensity (*λ*_ex_ = 492 nm, *λ*_em_ = 517 nm) of DMPE/DPPG/CHOL⊃CF vesicles (13 µM phospholipids in 10 mM Tris, 140 mM NaCl, pH 7.4) on addition of 300 µM of WR_7_ (red trace) or B_12_Br_12_^2−^ (black trace), demonstrating lysis caused by the former and retained membrane integrity in the presence of the latter. **g**, Compatibility of the carrier B_12_Br_12_^2−^ (blue, up to ca. 1 mM) with anionic liposomes (DMPE/DPPG/CHOL 1/2/1) in comparison to the strongly membrane-disrupting WR_7_ peptide (black) and pyrenebutyrate (**1**, red). *EC*_50_ for WR_7_ = 190 ± 50 µM and B_12_Br_12_^2−^ = 960 ± 100 µM. In **b**, **e** and **f**, Triton X-100 was added at *t* = 600 s to induce vesicle lysis and allow data normalization. Source data

For the iodinated cluster, B_12_I_12_^2−^, the addition of cluster alone led to an increase in fluorescence (Extended Data Fig. 1a, b). This observation indicated that this cluster, the largest and most chaotropic one, was disrupting the lipid membrane, which we independently confirmed by dynamic light scattering (DLS; Extended Data Fig. 1c) and an alternative vesicle leakage assay^36^ (carboxyfluorescein assay; Extended Data Fig. 1d, e). All other clusters studied herein preserved membrane integrity in the same sets of vesicular leakage experiments (Extended Data Fig. 1de), which ruled out the formation of pores or transitory bilayer disruption and encouraged us to investigate them as potentially viable membrane carriers. The parent B_12_H_12_^2−^, which is the smallest and least chaotropic in this series, did not show transport ﻿of WR_7_ in vesicles (Fig. 2b, pink trace). However, the chlorinated and brominated clusters, which are intermediary in size and chaotropicity, caused the desired time-resolved fluorescence responses, which signalled successful transport of the hepta-arginine peptide WR_7_ across the lipid membrane (Fig. 2b, green and red traces).

**Extended Data Fig. 2:**
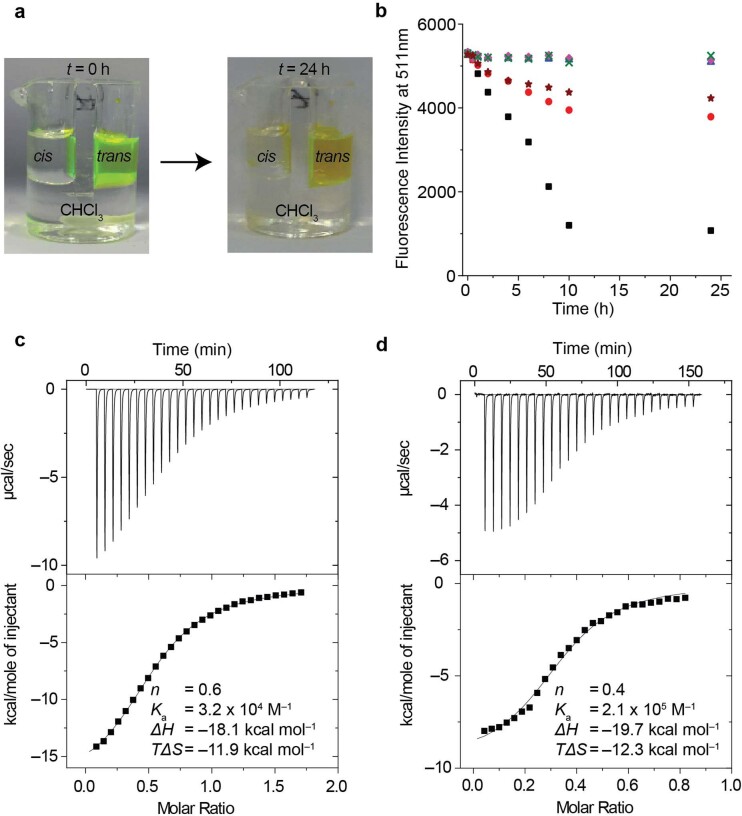
Mechanistic Investigations. **a**, Visualization of transport through a hydrophobic phase in U-tube experiments by taking photographs of the tri-phasic U-tube system at the start of an experiment (*t* = 0, left) and after 24 h (right). In the U-tube experiments, the *trans* compartment contained initially only the fluorescent HPTS dye and the *cis* compartment was administered with combinations of carrier (boron cluster), peptide cargo (WR_7_), and DPX, which serves as a quencher of HPTS. Although the *trans* phase is initially strongly fluorescent (left photograph), transport of DPX from the *cis* phase (10 mM Tris, 107 mM NaCl, pH 7.4) across the chloroform phase leads to a fluorescence quenching. **b**, HPTS fluorescence measured in the *trans* buffer of the U-tube (2 mM HPTS, 10 mM Tris, 107 mM NaCl, pH 7.4) on addition of the following agents to the *cis* buffer: 40 μM B_12_Br_12_^2−^ and 20 μM WR_7_ (▲); 20 mM DPX (×); 20 μM WR_7_ and 20 mM DPX (♦); 40 μM B_12_H_12_^2−^, 20 μM WR_7_, and 20 mM DPX (★); 40 μM B_12_Br_12_^2−^ and 20 mM DPX (•); 40 μM B_12_Br_12_^2−^, 20 μM WR_7_, and 20 mM DPX (■). The experiments demonstrate that there is (*i*) no fluorescence quenching in the absence of added DPX quencher (▲), (*ii*) no transport of the DPX quencher in the absence of carrier and peptide cargo (×), (*iii*) no transport of the DPX quencher in the presence of peptide cargo but in the absence of a carrier (♦), (*iv*) slow and inefficient transport of DPX in the presence of peptide cargo and the least efficient carrier, B_12_H_12_^2−^ (★), (*v*) faster and more efficient transport of DPX in the absence of peptide cargo and the very efficient carrier, B_12_Br_12_^2−^ (•), and (*vi*) the fastest and most efficient transport of DPX in the presence of peptide cargo and the very efficient carrier, B_12_Br_12_^2−^ (■). The experiments show that B_12_Br_12_^2−^ is itself an effective carrier of DPX, but that the carrier/peptide cargo system leads to the most effective transport. **c**,**d**, Calorimetric evidence for direct interaction of B_12_Br_12_^2−^ with peptide cargos as obtained from raw ITC data (top) and apparent reaction heats obtained from the integration of the calorimetric traces (bottom) for the sequential injections of 2 mM of B_12_Br_12_^2−^ into **c**, 250 µM WR_7_ and **d**, 450 µM WK_7_. 15% Error for *K*_*a*_ and ± 0.5 kcal mol^**−**1^ for Δ*H* and *T*Δ*S* (duplicate measurements). The calorimetric titrations show enthalpically driven non-stoichiometric intermolecular interactions between the cluster and the peptides. Source data

**Extended Data Fig. 3:**
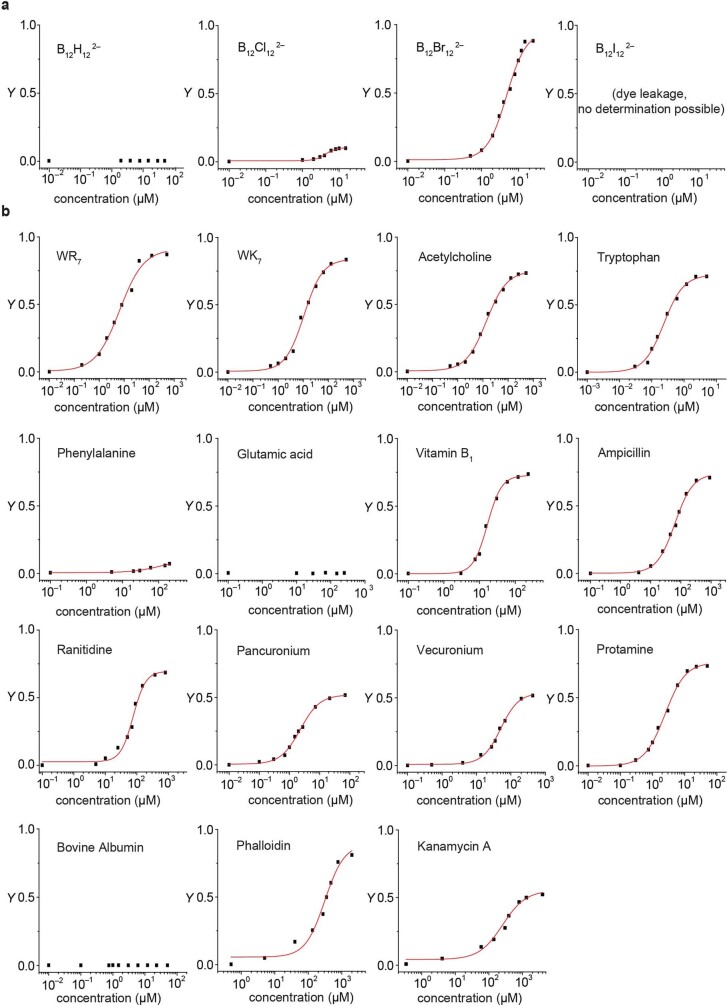
Transport activity. **a**, Transport activity, *Y*, in EYPC⊃HPTS/DPX vesicles as a function of cluster concentration at constant cargo concentration (WR_7_, 20 µM). **b**, Transport activity, *Y*, for B_12_Br_12_^2−^ (40 µM) in EYPC⊃HPTS/DPX vesicles as a function of the concentration of different cargo types; red lines correspond to the resulting fit with the Hill equation. Source data

U-tube transport experiments, across a bulk chloroform layer, unambiguously confirmed that the brominated cluster served as a non-covalent carrier across a hydrophobic bulk barrier (Extended Data Fig. 2a, b). To quantitatively characterize the transport efficiency of the new cluster carriers, the normalized fluorescence responses in the vesicle experiments (Fig. 2b) were plotted against cluster concentration to produce dose–response curves (Fig. 2c and Extended Data Fig. 3a). We extracted, by Hill analysis, the salient parameters: the maximal activity (*Y*_max_), the concentration needed to achieve 50% of *Y*_max_ (EC_50_) and the activator efficiency (*E*_a_)^18^. The data (Fig. 2b and Extended Data Table 1) showed that transport is activated for the chlorinated boron cluster but becomes most efficient for the brominated one, with *Y*_max_ = 95% and *E*_a_ = 6.1. This activity rivals the pyrenebutyrate (**1**) gold standard in the field (*E*_a_ ≈ 5)^18^, which we used throughout as a control.

From a molecular design point of view, it transpired that the transport activity critically depends on cluster size and type. B_12_H_12_^2−^, the least chaotropic cluster, is inactive in the vesicle experiments, although it shows an onset of transport in the U-tube experiments (Extended Data Fig. 2a, b). The largest and most chaotropic cluster, B_12_I_12_^2−^, appears to show too high activity (membrane affinity), as it causes membrane disruption even in the absence of cargo. The chlorinated cluster already shows sizeable transport activity, but the ‘sweet spot’ in this homologous cluster series is reached for B_12_Br_12_^2−^, the prototypical superchaotropic carrier.

## Broadband carrier characteristics

Once the brominated cluster, B_12_Br_12_^2−^, had emerged as an exponent of a new class of highly active synthetic membrane carriers, we focused on the scope of the compounds that can be transported^3^. For amphiphilic activators with anionic head groups, such as **1**, the synergy of the favourably oriented hydrogen bonds and permanent electrostatic interactions that the guanidinium groups can form with the carboxylates are considered prerequisites for efficient cargo translocation^19,37^. As a consequence, although transport of arginine-rich peptides is straightforward, that of lysine-rich peptides, which can be reversibly deprotonated to minimize charge repulsion, is already substantially more difficult^38^ and requires meticulous design of suitable receptors in the carriers^39^. As a challenge, we studied first the corresponding hepta-lysine (WK_7_) and then others, as alternative cargos for B_12_Br_12_^2−^ (Fig. 2d).

The cargo-screening experiments were conducted at a constant B_12_Br_12_^2−^ concentration of 40 µM, such that the extracted EC_50_ values and *Y*_max_ values can be used to define a relative scale of transport efficiency, *E*_t_, in which we set the value for the oligoarginine reference to 10. Surprisingly, without further carrier design, B_12_Br_12_^2−^ showed very similar transport efficiency for WK_7_ as for WR_7_ (Fig. 2d, e), which clearly breaks the trend for amphiphilic activation (for example, by **1**) that fails to trigger any signal of oligolysine membrane transport (Fig 2e). This non-canonical membrane translocation of the WK_7_ peptide by the boron cluster was further characterized by isothermal titration calorimetry (ITC), which confirmed an enthalpy-driven interaction of the B_12_Br_12_^2−^ cluster with both cationic peptides in homogeneous solution (Extended Data Fig. 2c, d). Intriguingly, the binding affinity of the boron cluster was even slightly stronger with the lysine peptide (WK_7_) than with the arginine one (WR_7_), which demonstrates that interactions other than the conventional ones (Coulombic, salt bridges or hydrogen bonding) are important contributors to the cluster–peptide affinity and translocation.

The dynamic, enthalpically driven binding of globular boron clusters to their cargo is a reflection of their generic affinity to hydrophobic matter (chaotropic effect)^8^. Accordingly, chaotropic carriers should be potentially capable of transporting not only cationic, but also neutral targets. This ‘broadband carrier’ hypothesis was validated with membrane-impermeable molecules of biological interest. Our targets included differently charged biomolecules (such as acetylcholine and amino acids), vitamins, antibiotics, neuromuscular blocking agents and proteins. Remarkably, B_12_Br_12_^2−^ transported many types of cargo, ranging from positive to non-charged and zwitterionic molecules, or from small ones, such as acetylcholine (molecular mass of 146 Da), to larger polypeptides, such as protamine (molecular mass of 4,500 Da), with the exception of the negatively charged molecules glutamate and albumin, for which no carrier–cargo charge attenuation can occur (Fig. 2d, Extended Data Fig. 3b and Extended Data Table 1). The very fast transport kinetics were in the range of seconds for most cargo types (Fig. 2f), which becomes competitive with uptake through membrane pores or channels^40^. The insensitivity towards the chemical nature of the diverse functional groups in the transported cargos (Extended Data Fig. 9) confirmed that the carrier activity of the boron clusters is not limited to residues that entertain salt bridges or specific intermolecular interactions, and is much less restrictive than for amphiphilic activators. Indeed, the prototype amphiphile **1** showed no activity for any of the newly introduced targets (Fig. 2f).

**Extended Data Fig. 4:**
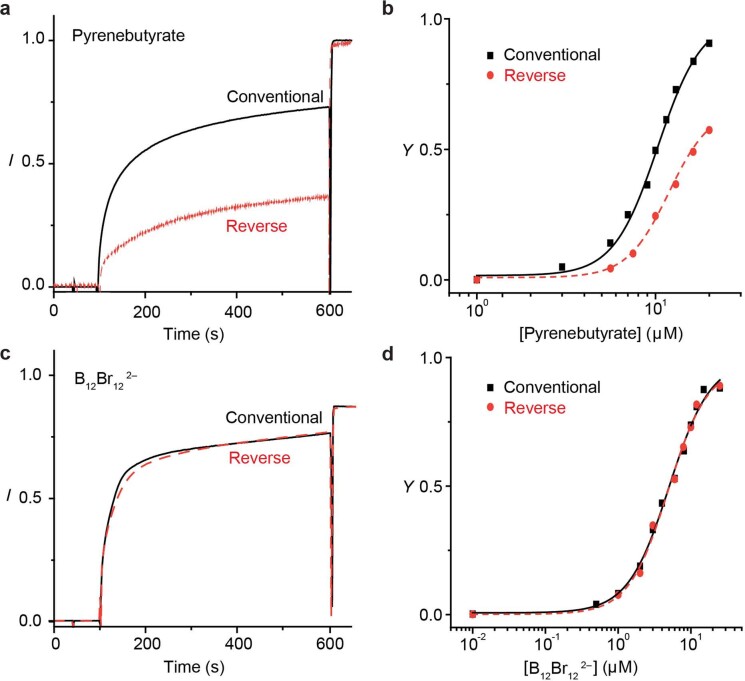
Membrane transport in vesicles in dependence on the sequence of cargo–carrier addition. **a**, **c**, Transport kinetics (120 μM pyrenebutyrate and 130 μM B_12_Br_12_^2−^) and **b**, **d**, dose–response curves of pyrenebutyrate (upper panels) and B_12_Br_12_^2−^ (bottom panels) for the transport of WR_7_ (20 μM) for a “conventional” (first carrier, then cargo, black, solid lines) vs a reverse addition (first cargo, then carrier, red, dashed lines). The transport kinetics (**a**) and efficiency (**b**) is strongly and adversely affected for pyrenebutyrate, but not for B_12_Br_12_^2−^ (**c**, **d**). Source data

Although the ITC experiments (see above) establish an intrinsic affinity between the boron clusters and the peptide cargos, this interaction does not lead to an irreversible adsorption, undesirable precipitation or the formation of nanoscale aggregates, as established by the absence of DLS effects. Note that the boron clusters are much more water soluble (high mM range)^8^ than most amphiphilic carriers or counterion activators. Consequently, their transport efficiency was also independent of the sequence of cluster/analyte addition, regardless of absolute transport kinetics (that is, rapid for acetylcholine or slow for pancuronium; Fig. 2g). This result is mechanistically meaningful, because all amphiphilic carriers described so far have been reported to require an incubation period to first enable insertion of the activator into the membrane^23^. Otherwise, if the peptide is added first and the amphiphilic carrier afterwards, cargo–carrier aggregation can occur before the carrier inserts into the membrane, which reduces the transport efficacy (Extended Data Fig. 4). In general, the binding processes of the superchaotropic clusters involve weak supramolecular interactions;^8^ these are characterized by a fast reversible binding to both the cargo and the membrane (Figs. 1c and 2a), which enables the efficient and rapid transport, as well as the broad cargo scope.

To better mimic biological membranes, complementary experiments in anionic vesicles were also performed. They showed that the di-anionic B_12_Br_12_^2−^ not only retained its transport activity in the anionic vesicles, but also largely outperformed the mono-anionic reference activator **1** (Fig. 2h), as was the case for the zwitterionic vesicles (Fig. 2c, f). Despite the excellent transport profile of B_12_Br_12_^2−^ in this assay, the boron cluster did not disrupt the anionic membranes at concentrations even one order of magnitude higher than those required for transport activation (Extended Data Fig. 1f, g). It can be concluded that the observed transport phenomena and the cargo scope in vesicle transport experiments are unique and a consequence of the shift from amphiphilic to superchaotropic carrier design.

## Membrane translocation in living cells

**Fig. 3:**
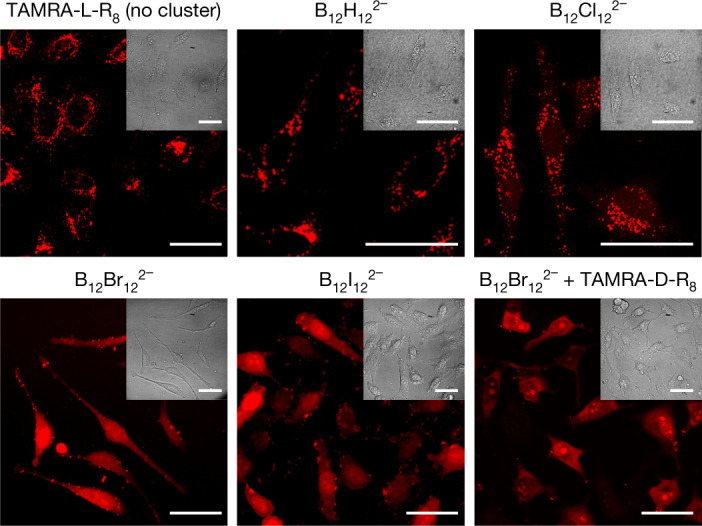
Cellular peptide uptake assisted by different boron clusters. HeLa cells were incubated with 1 µM TAMRA-R_8_ in the absence (‘no cluster’) or presence of 10 µM boron cluster diluted in HKR buffer for 1 h, washed with DMEM without phenol red and imaged by confocal fluorescence microscopy. The micrograph in the bottom right panel refers to incubation with the enantiomeric (hydrolysis-resistant) TAMRA-﻿D-R_8_ (1 μM) and B_12_Br_12_^2−^ (1 μM) for 30 min. Brightness and contrast of TAMRA-D-R_8_ were adjusted to avoid saturation; see Extended Data Fig. 6d for comparison with controls. Representative images of two biological replicates. Staining results were insensitive to incubation time (Supplementary Note 1). Scale bars, 50 μm.

Prompted by the success in vesicles, we decided to carry out transport experiments in living cells. Confocal fluorescence microscopy was used to study the potential of boron clusters to trigger membrane translocation of a model carboxytetramethylrhodamine-labelled R_8_ peptide (TAMRA-R_8_) in living cells (Fig. 3). At low concentrations (namely, 1 µM), cationic penetrating peptides usually remain trapped inside the endosomes of living cells^7,41^. In line with expectation, peptide-transport experiments in the absence of the clusters, or in the presence of those clusters that displayed no or low activity in the vesicle models, namely B_12_H_12_^2−^ and B_12_Cl_12_^2−^, showed only confocal micrographs with punctate fluorescence pointing to TAMRA-R_8_ trapped in endosomes (Fig. 3). However, in the presence of B_12_Br_12_^2−^, which had been found to be the most active in the liposomal experiments, diffuse peptide fluorescence was detected in the cytosol and the nucleus of the cells (Fig. 3), which signalled the desired carrier activity in the cells. The membrane-lytic iodinated cluster, B_12_I_12_^2−^, was also active but showed compromised cell morphology (see bright-field micrographs in Fig. 3).

**Extended Data Fig. 5:**
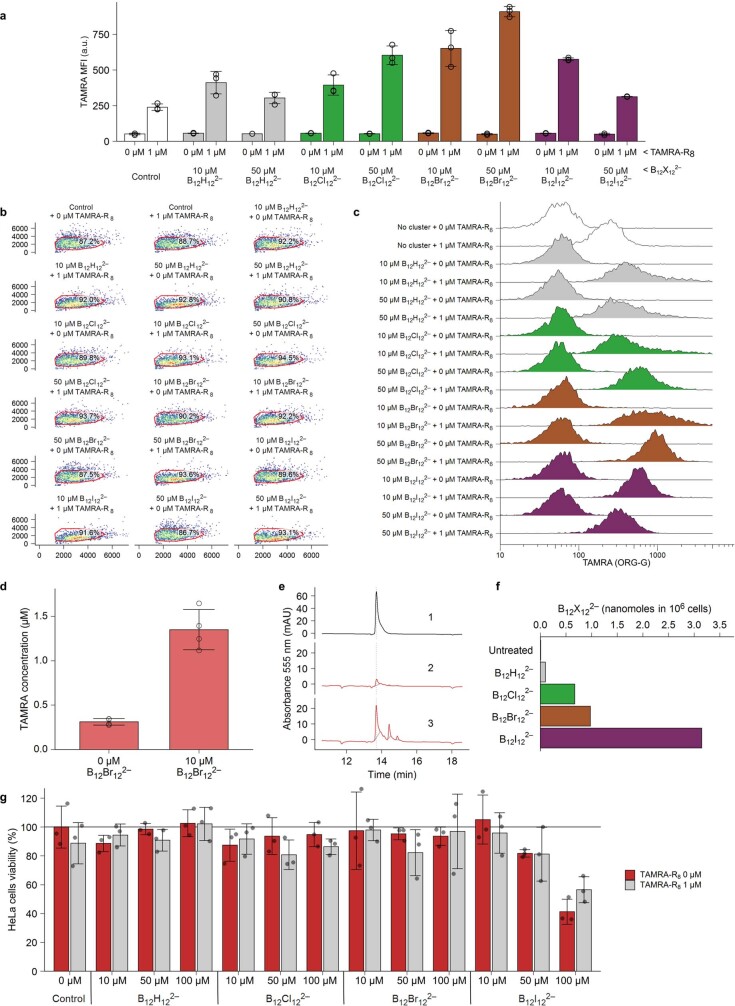
Cluster-mediated TAMRA-R_8_ cellular uptake, cellular cluster uptake and cluster toxicity. **a**, HeLa cells were incubated with 0 or 1 µM TAMRA-R_8_ and 10 or 50 µM of each cluster for 1 h and subsequently washed and trypsinized for flow cytometry analysis. Data are mean ± s.d. of the median fluorescence intensity of three replicates. **b**, Gating strategy for one of the replicates (axes: x = FSC, y = SSC). **c**, Normalized histograms showing TAMRA fluorescence in the gated population for one of the replicates. **d**, Cytosolic concentration of TAMRA-R_8_ in HeLa cells as calculated from extracts obtained after incubation with digitonin. Cells were incubated with 1 µM peptide, in the absence or presence of 10 µM cluster for 1 h. Cytosolic concentrations were calculated from the number of cells per well assuming a HeLa cell volume of 5 pL; the resulting cytosolic concentration in the presence of the cluster (1.35 ± 0.23 µM) was 4.4 times higher than the one obtained after incubation with the peptide alone (0.31 ± 0.04 µM). Background luminescence levels in the absence of the fluorescent peptide corresponded to apparent concentrations of less than 0.02 µM. Data are mean ± s.d. of four independent experiments, each with three technical replicates. **e**, HPLC analysis of TAMRA-R_8_ obtained after incubation with digitonin: pure peptide reference (1), HeLa cells incubated with 1 µM peptide (2), and HeLa cells incubated with 1 µM peptide and 10 µM cluster (3). Peak integration (as defined by the grey line) showed a ca. 5 times higher level of intact peptide in the presence of the cluster. **f**, Cellular uptake of boron clusters as determined through the content of atomic boron by ICP-MS. HeLa cells were lysed after 3 h incubation with 50 µM of each cluster diluted in HKR. **g**, Viability of HeLa cells incubated with the indicated clusters diluted in DMEM in the absence (red) or presence (grey) of 1 µM TAMRA-R_8_ for 1 h, washed and further incubated with complete medium containing 0.5 mg/mL of MTT. After medium removal and solubilization with DMSO, the absorbance at 570 nm was measured. Values were normalized to the untreated cell control (100%). Data are mean ± s.d. of three replicates. Source data

**Extended Data Fig. 6:**
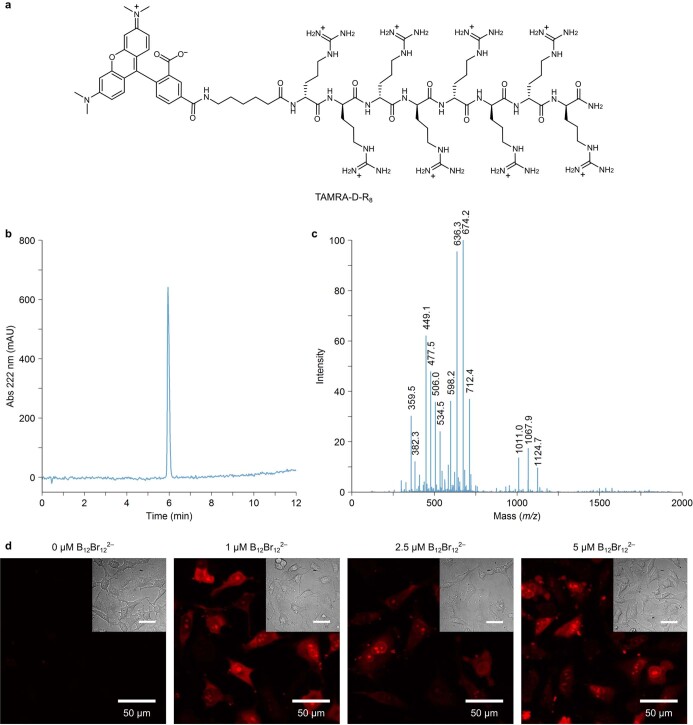
Characterization of the TAMRA-D-R_8_ enantiomeric peptide and its cellular uptake. **a**, TAMRA-D-R_8_ structure. **b**, RP-HPLC [Agilent SB-C18 column, H_2_O (0.1% TFA)/CH_3_CN (0.1% TFA) 95:5→5:95 (0→12 min)], *R*_t_: 5.96 min. **c**, ESI-MS of TAMRA-D-R_8_. **d**, Fluorescence images of HeLa cells obtained after incubation with 1 µM of TAMRA-D-R_8_ and the indicated concentrations of B_12_Br_12_^2−^ for 30 min and washing; bright-field images in insets; all scale bars are 50 µm; the second panel (1 µM) is also shown in Fig. 3 in main text. Representative images of two biological replicates. Note that the clusters did not affect uptake of the non-conjugated TAMRA probe (see Supplementary Note 3). Source data

Flow cytometry confirmed the enhancement of total TAMRA-R_8_ uptake in the presence of boron clusters in HeLa cells, with B_12_Br_12_^2−^ performing again as the most active carrier (Extended Data Fig. 5a–c). Digitonin fractionation experiments, followed by high-performance liquid chromatography (HPLC) analysis, confirmed a four to five times enhanced cytosolic uptake of the intact TAMRA-R_8_ peptide in the presence of the B_12_Br_12_^2−^ cluster (Extended Data Fig. 5d, e). In addition, confocal micrographs demonstrated an excellent cluster-mediated intracellular transport of the enantiomeric counterpart, TAMRA-D-R_8_, a hydrolysis-resistant peptide that remains fully intact during the transport experiment (Fig. 3 and Extended Data Fig. 6). Complementary inductively coupled plasma–mass spectrometry (ICP-MS) experiments showed that the boron clusters accumulated into cells following their chaotropic character (Extended Data Fig. 5f). Nevertheless, low cellular toxicity in the MTT assay was confirmed in the transport experiments for the clusters, even at 100 µM, except for the iodinated derivative (Extended Data Fig. 5g).

## New cargo types and biological activity

Beyond enhancing membrane translocation of cationic peptides in vesicles and living cells, boron clusters also show a broad scope of accessible cargo types, including neutral hydrophilic molecules (Fig. 2d, f and Extended Data Table 1). One of the successfully transported non-charged hydrophilic cargos in vesicles was phalloidin, a rigid bicyclic heptapeptide that has been long known in cell biology for its ability to bind to F-actin of the cytoskeleton^42^. At the same time, phalloidin is notorious for resisting internalization, and cell fixation and membrane permeabilization are traditionally used for cytoskeleton labelling purposes^43^. Phalloidin delivery in living cells has been explored by covalent modifications of the cargo itself with polycationic dendrimers^43^, or by membrane-disrupting strategies, such as optoporation^44^, the addition of pore-forming toxins^45^ or redox-sensitive polymer-based strategies^46^. However, a routine strategy for phalloidin delivery, such as one based on the addition of a low molecular mass non-covalent carrier, has been elusive.

**Fig. 4:**
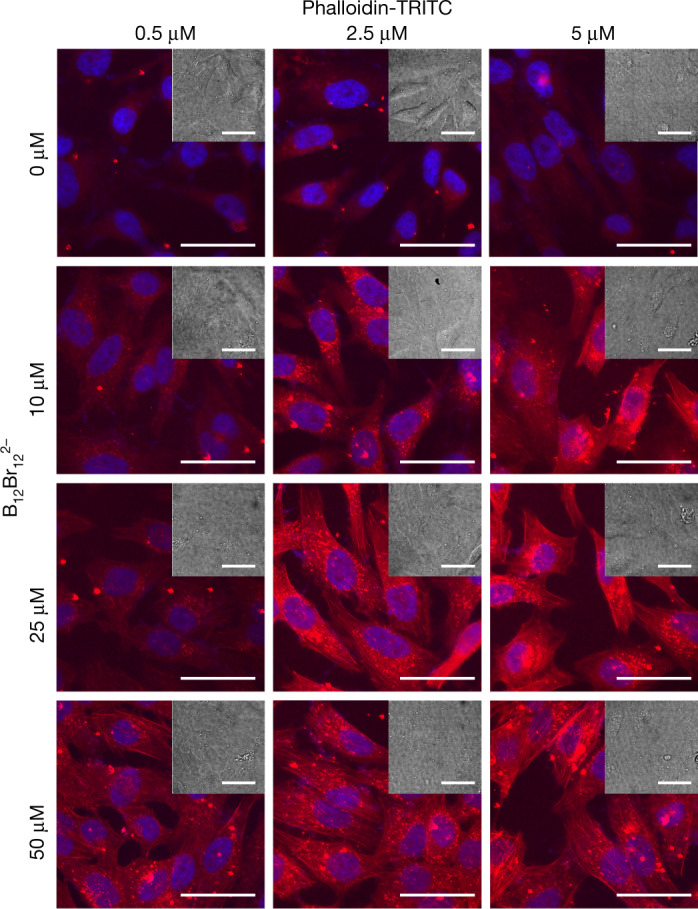
B_12_Br_12_^2−^-assisted phalloidin-TRITC transport into living HeLa cells. Cells were incubated with 0.5, 2.5 and 5 µM phalloidin-TRITC (red, from left to right) in the absence (top row) and presence of different concentrations of B_12_Br_12_^2−^ cluster (10, 25 and 50 µM; second, third and fourth rows, respectively) in HKR buffer for 3 h, subsequently stained with Hoechst (blue), washed for 5 min with 0.1 mg ml^−1^ heparin in HKR buffer and imaged by confocal fluorescence microscopy; bright-field images in insets. Representative images of two biological replicates. Staining results were insensitive to sequence of addition (Supplementary Note 2). Scale bars, 50 μm.

**Extended Data Fig. 7:**
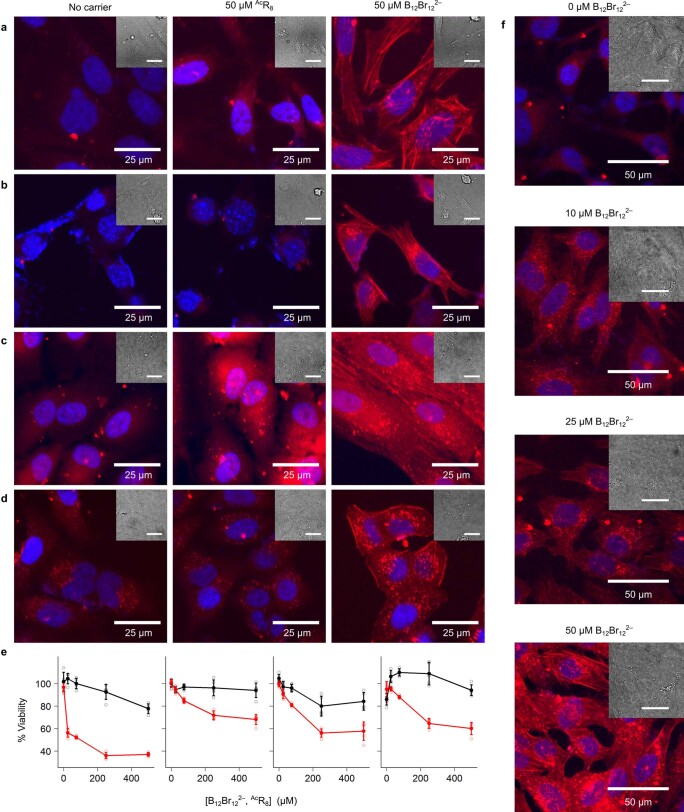
B_12_Br_12_^2−^-assisted phalloidin-TRITC transport into living cells. **a**, HeLa, **b**, GT1-7, **c**, ARPE-19 and **d**, A549. Cells were incubated with 7 µM phalloidin-TRITC (red) in the absence (left) and presence of 50 µM ^Ac^R_8_ (middle) or 50 µM of B_12_Br_12_^2−^ cluster (right) in HKR buffer for 3 h, subsequently stained with Hoechst (blue), washed with DMEM without phenol red, and imaged by confocal fluorescence microscopy; bright-field images in insets. Micrographs from each cell line were processed independently, experiments were performed twice. **e**, MTT viability assay of the corresponding cell lines (HeLa, GT1-7, ARPE-19 and A549, from left to right) at different concentrations (0, 25, 75, 250, and 500 µM) of B_12_Br_12_^2−^ (black) or ^Ac^R_8_ (red); data are mean ± s.d. of five measurements. **f**, Confocal images of HeLa cells after incubation with 500 nM phalloidin-TRITC and different concentrations B_12_Br_12_^2−^; the images correspond to those of Fig. 4 in the main text, but are shown with increased brightness to facilitate observation of the actin filaments. Representative images of two biological replicates. All scale bars are 25 µM, except for panel f (50 µm). Source data

Phalloidin-TRITC transport experiments with living HeLa cells showed that B_12_Br_12_^2−^ triggered its direct membrane passage to the cytosol and provided excellent staining of the F-actin target even at 500 nM cargo concentration (Fig. 4 and Extended Data Fig. 7f). This protocol was transferable to GT1-7﻿ mouse hypothalamic GnRH neuronal cells, human retinal pigmentary epithelial cells ARPE-19 and adenocarcinoma human alveolar basal epithelial cells A549 (Extended Data Fig. 7a–d). By contrast, when attempting to use the prototypical octa-arginine-penetrating peptide ^Ac^R_8_ as an alternative non-covalent carrier for the same set of cell lines, only trace levels of cytosolic phalloidin were observed in all cell lines (Extended Data Fig. 7a–d). Under the transport experimental conditions, the superchaotropic cluster B_12_Br_12_^2−^ also showed a lower toxicity than its penetrating peptide competitor ^Ac^R_8_ (Extended Data Fig. 7e).

**Extended Data Fig. 8:**
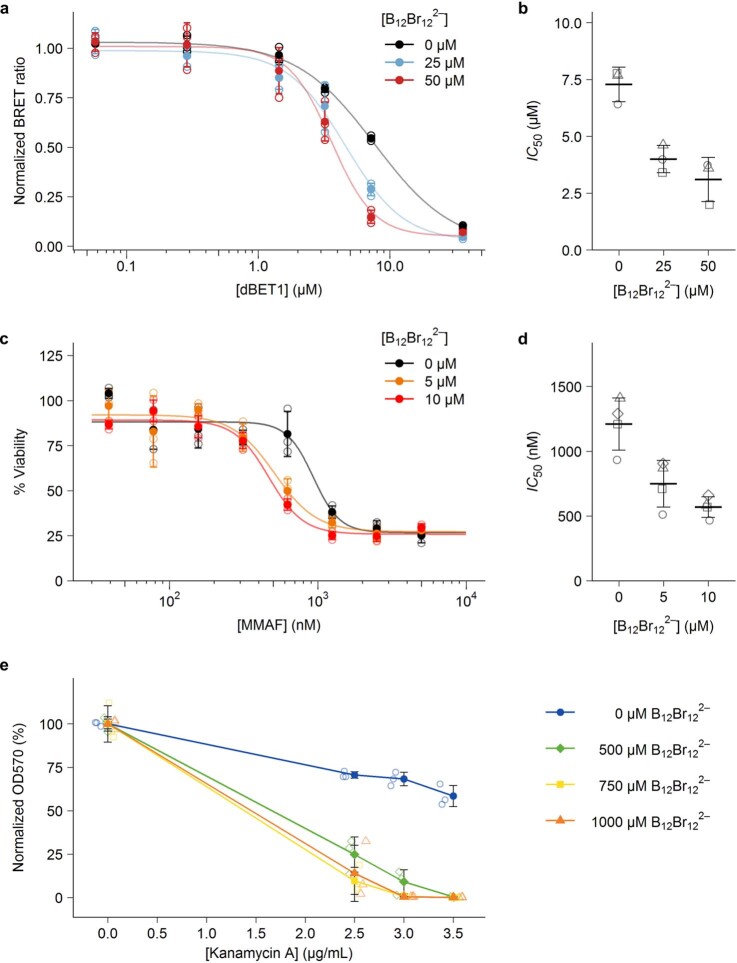
Boron cluster-induced enhancement of biological activity. **a**, Dose–response experiment for dBET1 binding to the CRBN E3 Ubiquitin ligase, quantified with the CRBN NanoBRET target engagement assay, in the presence of 0, 25 and 50 μM B_12_Br_12_^2−^. In this assay, HEK293 cells are transfected with a plasmid expressing a CRBN-NanoLuc fusion protein for 24 h and subsequently incubated with a labelled tracer molecule (0.5 µM) and the test compounds. dBET1 acts as a competitive inhibitor and decreases the NanoBRET signal between CRBN-NanoLuc and the tracer. Solid points indicate mean ± s.d. of three technical replicates. Values were normalized to the controls without dBET1, as indicated in the Methods section. **b**, Corresponding *IC*_50_ values calculated with the CRBN Target Engagement assay. Cross-bars indicate mean ± s.d. of three independent experiments (two with technical triplicates, one with technical duplicates); values obtained in each experiment are represented by different shapes. **c**, Viability of HeLa cells after incubation with different doses of MMAF in the presence of 0, 5 and 10 μM B_12_Br_12_^2−^. Solid points indicate mean ± s.d. of three technical replicates. **d**, Corresponding *IC*_50_ values of MMAF. Cross-bars indicate mean ± s.d. of four independent experiments, each with technical triplicates; each experiment is represented by a different shape. **e**, *E. coli* Top10 viability in the presence of different concentrations of kanamycin A monosulphate (0–3.5 μg/mL) and B_12_Br_12_^2−^ (0–1,000  μM) in LB broth at 37 °C; data are mean ± s.d. of triplicate measurements. Source data

**Extended Data Fig. 9:**
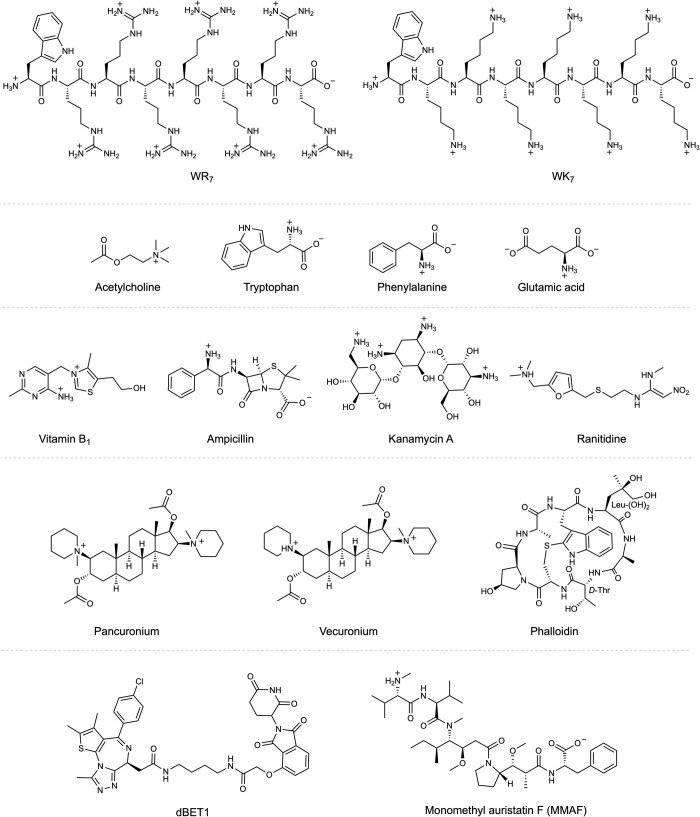
Chemical structures of the low molecular weight analytes studied. Predominant charges at neutral pH are shown.

Proteolysis targeting chimeras (PROTACs) are small molecules with a bright future as the next generation of drugs for the removal of specific unwanted proteins. We tested whether the use of boron clusters could contribute to enhancing the activity of dBET1, a well-characterized PROTAC that is known for its undesirable low permeability^47^ and that should fall within the potential cargo scope of B_12_Br_12_^2−^ (neutral, molecular mass of 785 Da). The internalization of the PROTAC in the absence and presence of the cluster was assessed by its ability to bind to the Cereblon E3 ligase by using the NanoBRET TE intracellular E3 ligase assay (Extended Data Fig. 8a, b). In this assay, a cluster-enhanced uptake of dBET1 was indeed observed (factor 2–3 decrease in half-maximum inhibitory concentration (IC_50_) value), which illustrates the versatility of the new carriers. We also demonstrated the cluster-mediated intracellular transport of monomethyl auristatin F (MMAF, zwitterionic, molecular mass of 732 Da), an antineoplastic drug with considerably lower permeability in comparison to other auristatins^48^. For this bioactive cargo also, the B_12_Br_12_^2−^ cluster was found to effectively reduce its IC_50_ value by more than a factor of 2, as assessed through the viability of HeLa cells (Extended Data Fig. 8c, d).

Delivery of antibiotics is another area in which novel carrier concepts are intensively sought, and our vesicle studies demonstrated transport of ampicillin and kanamycin A by the prototype chaotropic cluster carrier, B_12_Br_12_^2−^ (Fig. 2d, f). As a proof-of-principle, we investigated its potential to reduce the minimum inhibitory concentration of kanamycin A, an aminoglycoside antibiotic. Aminoglycosides function by binding to the bacterial 30S ribosomal subunit; consequently, effective passage through the cell wall and plasma membrane is essential for aminoglycosides to reach their intracellular targets^49^. The antibiotic resistance of the Gram-negative *Escherichia coli* Top10 strain to the action of kanamycin A (3.5 µg ml^−1^) was investigated in the absence and presence of B_12_Br_12_^2−^ (Extended Data Fig. 8e). In the absence of cluster, *E. coli* retained viability (60%), but in the presence of the cluster carrier kanamycin A showed potent antibacterial activity (<1% viability). The fact that incubation with the cluster alone did not affect bacterial survival up to 1 mM demonstrates that the combination of both, antibiotic and carrier, is essential to prompt the biological response.

The combined transport experiments and the successful functional delivery of different bioactive cargos demonstrate that boron clusters, and prominently B_12_Br_12_^2−^, are able to transport the intact agents through the cellular bilayer membrane, at physiologically relevant concentrations, and to induce the corresponding enhanced biological effects. Nevertheless, this route is still preliminary, especially for the delivery of (bio)macromolecules.

## Discussion

The rational design of effector molecules with biological activity is constrained by physicochemical concepts traditionally derived from the observation of molecules or processes found in nature. The model of amphiphilicity has, in particular, governed the design of membrane carriers for the last 50 years^1^. The hydration–thermochemical properties of superchaotropic anions differ from those of hydrophobic or amphiphilic solutes and, on a continuous scale of solvation in water, they fall in between hydrophobic ions and conventional chaotropes^8^. The chaotropic effect, that is, the interaction of superchaotropes with hydrophobic phases, surfaces and concavities, is enthalpically driven, by a combination of desolvation effects as well as by strong dispersion interactions, and it differs from the thermochemical signature of the classical hydrophobic effect, which is entropically driven.

Our results introduce superchaotropic globular boron cluster anions as a chemically distinct class of membrane carriers. The clusters obviate the traditional amphiphilic transport mechanism in that they operate by a direct chaotrope-mediated translocation (Fig. 1c), as experimentally confirmed by vesicle, ITC, U-tube and cellular assays for different cargos (Fig. 2d). As reported here, the enthalpy-driven complexation and non-canonical transport of cationic peptides with either guanidinium or ammonium moieties (Fig. 2e), the retained transport regardless of the sequence of cargo/carrier addition (Fig. 2g), the independence of cargo uptake on membrane charge (Fig. 2h) and the efficient translocation of selected neutral hydrophilic cargos (Fig. 2d, f) in vesicles and cells distinguish these new globular anionic carriers. In this complementary membrane transport concept, the low dehydration penalty and the strong dispersion interactions of superchaotropic clusters minimize the repulsion between the hydrophilic molecules and the membrane barrier and, thus, enable the direct passage of a broad range of cargos across lipid membranes.

Owing to the biocompatibility^29^ and broad cargo scope of the boron clusters, as well as the enhancements in bioactivity of different molecular effectors, ideas for therapeutic amino acid or peptide delivery, as well as pharmaceutical applications, come to mind, for example, in topical drug delivery. From a chemical viewpoint, other boron cluster congeners and chemically modified clusters could expand the cargo selectivity of this new class of membrane carriers^50^.

## Methods

### Chemicals, peptides and cell lines

Boron clusters (as sodium salts) were from Katchem, streptomycin sulfate and kanamycin A monosulfate both from Sigma, MMAF from Carbosynth and dBET1 from Cayman Chemicals. Peptides (WR_7_ and WK_7_) were custom-made by Biosyntan in >98% purity as confirmed by HPLC and MS. TAMRA-R_8_ was synthesized by solid-phase peptide synthesis, as reported^51^. HeLa, HEK293, ARPE-19 and A549 cells were obtained from ATCC, and GT1-7 cells were obtained from Millipore. HeLa, HEK293, GT1-7 and A549 cells were maintained in DMEM, and ARPE-19 in DMEM/F-12, in all cases supplemented with 10% FBS and 1% penicillin–streptomycin–glutamine mix, at 37 °C, 5% CO_2_ and 95% humidity.

### Vesicle preparation

A thin lipid film was prepared by evaporating a lipid solution with a stream of nitrogen and then dried in vacuo overnight. For the zwitterionic vesicles, 25 mg EYPC in 1 ml of CHCl_3_ was used, and for the anionic vesicles, DMPE/DPPG/CHOL (4.4/10.4/2.6 mg, 1/2/1 molar ratio) in a 1:1 mixture of CHCl_3_ and MeOH (1 ml) was used. To prepare the lipid⊃HPTS/DPX vesicles (where ⊃ indicates encapsulation), the dry film was rehydrated (for 30 min at ambient temperature for EYPC, and for 60 min at 55 °C for DMPE/DPPG/CHOL) with 1 ml buffer (5 mM HPTS, 16.5 mM DPX, 10 mM Tris, 72 mM NaCl, pH 7.4) and subjected to 10 freeze–thaw cycles and extrusions (15 times) through a polycarbonate membrane (pore size 100 nm). Extravesicular components were eluted by size exclusion chromatography (NAP-25 column Sephadex G-25 DNA grade) with 10 mM Tris, 107 mM NaCl, pH 7.4 (ambient temperature for EYPC, 65 °C for DMPE/DPPG/CHOL). The lipid⊃CF vesicles were prepared analogously, except for the types of rehydration/﻿elution buffers, which were 50 mM CF, 10 mM HEPES, pH 7.5/10 mM HEPES, 107 mM NaCl, pH 7.5 for EYPC⊃CF and 100 mM CF, 10 mM Tris, pH 7.4/10 mM Tris, 140 mM NaCl, pH 7.4 for DMPE/DPPG/CHOL⊃CF.

### Transport experiments in HPTS/DPX vesicles

EYPC vesicle stock solutions (5–8 µl) were diluted with buffer (10 mM Tris, 107 mM NaCl, pH 7.4) in a disposable plastic cuvette and gently stirred (total volume 2,000 µl, final lipid concentration 13 µM). HPTS fluorescence was monitored at wavelength *λ*_em_ = 511 nm (*λ*_ex_ = 413 nm) as a function of time after addition of boron clusters at 50 s, analyte at 100 s and Triton X-100 (24 µl, 1.2% wt/vol) at 600 s, the latter to lyse the vesicles, for calibration. Fluorescence intensities were normalized to fractional emission as *I*(*t*) = (*I*_t_ − *I*_0_)/(*I*_∞_ − *I*_0_), where *I*_0_ = *I*_t_ before cluster addition and *I*_∞_ = *I*_t_ after lysis. For Hill analysis, *I*_t_ before lysis was defined as transport activity, *Y*, and plotted against cluster (or analyte) concentration, *c*, and fitted to the Hill equation *Y* = *Y*_0_ + (*Y*_max_ − *Y*_0_)/(1 + (EC_50_/*c*)^*n*^), to give the activity in the absence of cluster, *Y*_0_, the maximal activity, *Y*_max_, the concentration needed to achieve 50% of maximal activity, EC_50_, and the Hill coefficient, *n*.

### Activator efficiency

In the activator measurements, in which different activators were tested with the same cargo, the activator efficiency (*E*_a_) is determined from their ability to activate the transport of an impermeable cargo molecule and is characterized by *Y*_max_, its maximal activity, and EC_50_, the effective activator concentration. A potent activator reaches high *Y*_max_ at low EC_50_. To reflect both factors, the activator efficiency is defined as *E*_a_ = *Y*_max_ × (pEC_50_/*f*_a_), where pEC_50_ is the negative logarithm of EC_50_. To enable comparison with literature studies, which aimed for a scale of *E*_a_ values from 0 to 10 (ref. ^18^), the scaling factor *f*_a_ was set to 20.6.

### Transport efficiency

In the transport measurements, in which different types of cargo were tested with the same activator, the transport efficiency (*E*_t_) reports on the sensitivity of the cargo for being transported and is described by *Y*_max_, the maximal activity, and EC_50_, the effective cargo concentration. An easily accessible cargo reaches high *Y*_max_ at low EC_50_. The transport efficiency is defined as a composition of both parameters according to *E*_t_ = *Y*_max_  × (pEC_50_/*f*_t_), where pEC_50_ is the negative logarithm of EC_50_. The scaling factor *f*_t_ was deliberately set to 19.8 such that the *E*_t_ value of the reference compound, WR_7_, equals 10.0, also in an effort to set up a scale from 0 to 10.

### Leakage experiments in CF vesicles

For leakage experiments with the lipid⊃CF vesicles, stock solutions (6 µl) were diluted with the respective buffer in a disposable plastic cuvette and gently stirred (total volume 2,000 µl, final lipid concentration 13 µM). CF fluorescence was monitored at *λ*_em_ = 517 nm (*λ*_ex_ = 492 nm) as a function of time after addition of the respective activating or disrupting agent (cluster, WR_7_ or pyrenebutyrate) at 50 s, and Triton X-100 (24 µl 1.2% (wt/vol)) at 600 s, the latter to lyse the vesicles, for calibration. Fluorescence intensities were normalized to fractional emission intensity as *I*(*t*) = (*I*_t_ −* I*_0_)/(*I*_∞_ − *I*_0_), where *I*_0_ = *I*_t_ before disrupting agent addition and *I*_∞_ = *I*_t_ after Triton X-100 lysis. For Hill analysis of the data for the DMPE/DPPG/CHOL⊃CF vesicles, *I*_t_ before Triton X-100 lysis was defined as membrane-disrupting activity, *Y*, and plotted against disrupting agent concentration, *c*, and fitted to the Hill equation *Y* = *Y*_0_ + (*Y*_max_ − *Y*_0_)/(1 + (EC_50_/*c*)^*n*^), to give *Y*_0_, *Y*_max_, EC_50_ and *n*.

### U-tube transport experiments

The U-tubes were home-made, similarly to those of Rebek and co-workers^52^ and Matile and co-workers^19^, and consisted of a small beaker with a central glass barrier separating the two aqueous phases, namely *cis* (sampling phase) and *trans* (receiving phase), but enabling the placement of an interfacing chloroform layer below the *cis* and *trans* phases. A 3 ml portion of CHCl_3_ was located in the U-tube and 1 ml of the *cis* and *trans* phases were added. The organic phase was stirred at 700 r.p.m. at room temperature. Aliquots (20 μl) from the aqueous *trans* phase were taken at different times, diluted to 450 μl with buffer (10 mM Tris, 107 mM NaCl, pH 7.4) and measured by fluorescence.

### Isothermal titration calorimetry

All experiments were performed in a VP-ITC MicroCalorimeter from MicroCal, at atmospheric pressure and 25 °C. Solutions were degassed and thermostated before the titration experiments in a ThermoVac accessory. A constant volume of B_12_Br_12_^2−^ (10 µl per injection) was injected into the peptide solution (WR_7_ or WK_7_) in water to determine the apparent binding affinity of B_12_Br_12_^2−^ with the peptides. Dilution heats were determined by titration of B_12_Br_12_^2−^ into water and subtracted from the reaction heat. The neat reaction heat was fitted with Origin v.7.0 and v.8.0 software by using a one-set-of-sites model to obtain the complex stability constant (*K*_a_) and molar reaction enthalpy (Δ*H*º). The free energy (Δ*G*º) and entropy changes (Δ*S*º) were obtained according to the relation Δ*G*º = −R*T*ln*K*_a_ = Δ*H*º − *T*Δ*S*º.

### Dynamic light scattering

DLS experiments were carried out on a Malvern Instruments DTS Nano 2000 Zeta-Sizer. Note that DLS measurements of the combinations of the B_12_Br_12_^2−^ clusters with the different cargos did not show any detectable signal of particles of DLS-measurable size.

### Cell culture and confocal imaging

For confocal microscopy studies, HeLa cells were seeded the day before on a µ-Slide 8 well (ibidi) at a density of 30,000 cells per well. The clusters and/or peptides were diluted in HKR buffer (5 mM HEPES, 137 mM NaCl, 2.68 mM KCl, 2.05 MgCl_2_, 1.8 CaCl_2_, pH 7.4) and added to the cells previously washed with HKR. HeLa cells were incubated with TAMRA-R_8_ (1 µM) and dodecaborate clusters in HKR buffer for 1 h at 37 °C, 5% CO_2_, washed with DMEM without phenol red and immediately imaged using Fusion software (Andor) with a Dragonfly spinning disc confocal microscope mounted on a Nikon Eclipse Ti-E and equipped with an Andor Zyla 4.2 PLUS sCMOS digital camera. For the phalloidin delivery studies, HeLa, GT1-7, ARPE-19 and A549 cells were incubated with phalloidin-TRITC and the boron cluster for 3 h and subsequently the nuclei were stained with 1 µM Hoechst 33342 for 20 min right before imaging. Images were processed with FIJI v. 2.1.0/1.53e (ref. ^53^).

### Cell viability assay

For MTT assays in the presence of the clusters and TAMRA-R_8_, HeLa cells were seeded the day before in 96-well plates at 10,000 cells per well. Cells were incubated with the clusters dissolved in DMEM, in the presence or absence of 1 µM TAMRA-R_8_ for 1 h. The incubation mixtures were replaced with DMEM + 10% FBS + 0.5 mg ml^–1^ MTT. For the viability assays in the presence of B_12_Br_12_^2−^ or R_8_, HeLa, GT1-7, ARPE-19 and A549 cells were seeded the day before in 96-well plates at 6,000 cells per well. Cells were incubated with B_12_Br_12_^2−^ or R_8_ dissolved in HKR buffer for 3 h and, thereafter, incubated for 24 h with complete medium before incubating with complete medium and 0.5 mg ml^−1^ MTT. For viability studies in the presence of MMAF, HeLa cells were incubated with MMAF and B_12_Br_12_^2−^ diluted in DMEM (without serum or antibiotics) for 3 h. Cells were washed with 0.1 mg ml^−1^ heparin and further incubated for 21 h in complete medium and 2 h in complete medium containing 0.5 mg ml^−1^ MTT. For all types of assays, after 2 h of incubation, the medium was carefully removed, and formazan crystals dissolved by addition of DMSO. The absorbance at 570 nm was measured with a plate reader (Tecan Infinite F200Pro) and the data normalized to the value of untreated cells (100% viability). Data were analysed with R (v. 4.0.3)^54^.

### Kanamycin A delivery in *E. coli*

A preculture of *E. coli* Top10 cells was incubated overnight in LB medium with 50 µg ml^−1^ streptomycin sulfate. The following day, 10^3^–10^4^ colony forming units per ml were grown in Costar cell culture 96-well plates in the presence of different concentrations of kanamycin A monosulfate (0, 2.5, 3 or 3.5 µg ml^−1^) and B_12_Br_12_^2−^ (0, 500, 750 or 1,000 µM) in LB medium without streptomycin at 37 °C in a shaking incubator. After 18 h, the optical density at 570 nm, as an indicator of bacterial growth, was measured with a Tecan Infinite F200Pro microplate reader. Data were normalized for each concentration of B_12_Br_12_^2−^ relative to the control condition without antibiotic.

### CRBN target engagement assay

This assay was performed according to the protocol by the manufacturer (Promega), with the required adaptation for carrier addition. HEK293 cells were co-transfected with the plasmids for NanoLuc-CRBN and DDB1 expression using Lipofectamine 2000. Cells were trypsinized, resuspended in Opti-MEM I at 200,000 cells per ml, and 34 μl dispensed on a white, non-binding surface plate (Corning). dBET1 serial dilutions were prepared in DMSO at 1,000× concentration, and further diluted in Opti-MEM I to 20×. B_12_Br_12_^2−^ was diluted to 20× in Opti-MEM I and mixed 1:1 with the dBET1 solutions. A 2 μl portion of NanoBRET target tracer CRBN reagent (final concentration, 0.5 μM) and 4 μl of the dBET1/B_12_Br_12_^2−^ mixtures were added to the cells, which were incubated for 2 h at 37 °C. Complete substrate-plus-inhibitor solution was prepared and bioluminescence resonance energy transfer (BRET) was measured with a Tecan Infinite 200Pro plate reader (filters Blue2 and Red; integration time of 1 s). Background correction was carried out by subtracting the signal of a sample without tracer. Values of each B_12_Br_12_^2−^ concentration series were normalized to the BRET readout of the controls without dBET1. Data were analysed with R (v.4.0.3)^54^.

### Cytosolic TAMRA-R_8_ concentration

Cytosolic extracts were obtained according to a previously described protocol^55^ by incubation with digitonin, a steroidal saponin that preferentially permeabilizes cholesterol-rich membranes, such as the plasma membrane, with minor effects on intracellular membranes. Briefly, HeLa cells were seeded at 260,000 cells per well in six-well plates, washed the next day twice with HKR, incubated with 1 µM TAMRA-R_8_ (the L enantiomer) in the presence or absence of 10 µM B_12_Br_12_^2−^ for 1 h, washed twice with HKR, three times with 2 mg ml^−1^ heparin in HKR and once with ice-cold PBS containing calcium and magnesium. Cells were incubated on ice with 600 µl of 35 µg ml^−1^ digitonin in PBS Ca/Mg for 10 min, the supernatant with the cytosolic fraction collected and cells washed with 200 µl of PBS Ca/Mg, combining this supernatant with the previous extract. The non-cytosolic fraction was collected by incubation of the cells with 800 µl of 1% Triton X-100 in PBS. TAMRA fluorescence of the extracts was determined in a plate reader (Tecan Infinite 200Pro, *λ*_ex_ = 555 nm, *λ*_em_ = 585 nm) and concentrations were calculated by using a calibration curve with serial dilutions of TAMRA-R_8_. For the complementary HPLC analysis, phosphate buffer was replaced by TBS (20 mM Tris–HCl, pH 7.2, 150 mM NaCl, 0.5 mM CaCl_2_, 0.5 mM MgCl_2_) and digitonin extraction was performed as indicated above. An aliquot of these extracts was used for β-hexosaminidase activity determination. Cytosolic extracts were lyophilized and resuspended in 1:10 volumes of H_2_O:CH_3_CN 1:1 with 1% TFA, and analysed by HPLC (RP-HPLC Agilent Luna 5U C18 100 Å, H_2_O (0.1% TFA)/CH_3_CN (0.1% TFA) 100:0 (0→5 min); 100:0→5:95 (5→20 min)) by monitoring the 555-nm absorbance of the TAMRA chromophore.

The quality of fractionation was assessed by lysosomal β-hexosaminidase activity, using 4-nitrophenyl 2-acetamido-2-deoxy-β-d-glucopyranoside as substrate. Briefly, 20 µl of extract was incubated with 80 µl of 7.5 mM substrate in 100 mM citrate buffer, pH 4.7, for 40 min at 37 °C, and the reaction was stopped by addition of 200 µl of 0.2 M Tris solution. Absorbance at 405 nm was measured in a plate reader. As blank, wells containing only the substrate were used. The enzymatic activities were found to be 3.2 ± 2.0% in the presence of the peptide and 5.4 ± 1.0% in the presence of peptide and cluster, confirming a high purity of the cytosolic fractions.

### ICP-MS

HeLa cells, seeded at 260,000 cells per well in six-well plates the day before, were washed with HKR and incubated for 3 h with 2.5 ml per well of 50 µM of each boron cluster diluted in HKR. Cells were washed with HKR containing 0.1 mg ml^−1^ heparin, twice with HKR and subsequently lysed with concentrated nitric acid (69% HNO_3_). Cells from nine wells were pooled for each sample. Lysates were diluted before analysis by ICP-MS in an Agilent 7700x equipped with a MicroMist glass low-flow nebulizer, a double-pass spray chamber with a Peltier system (2 °C) and a quartz torch. A calibration curve for the element boron (B) between 10 and 1,000 μg l^−1^ was prepared with the element germanium (Ge) as internal standard. The ICP-MS instrument parameters were as follows: RF power, 1,550 W; sample depth, 8﻿ mm; carrier gas flow, 1.1 l min^−1^; nebulizer pump speed, 0.1 r.p.s.; S/C temperature, 2 °C. Other parameters were set as follows: extract 1, 0; extract 2, −175; omega bias, −100; omega lens, 12.6; cell entrance, −40; cell exit, −60; deflect, 0.4; plate bias, −60; QP bias, −15; OctP RF, 180; OctP bias, −18; He gas, 3.6; discriminator, 4.5 mV; analogue HV, 1,730 V; pulse HV, 954 V.

### Flow cytometry

HeLa cells were seeded at 10,000 cells per well in 96-well plates. The next day, they were incubated for 1 h with the indicated compounds diluted in HKR. Cells were subsequently washed for 5 min with HKR containing 0.1 mg ml^−1^ heparin, washed again with HKR and trypsinized. Trypsin was neutralized with PBS containing 2% FBS and 5 mM EDTA. TAMRA fluorescence was excited with a green laser (532 nm) and measured on a Guava easyCyte BG HT collecting the emission at 620/52 nm (Orange-G channel) and using InCyte v.3.2 (GuavaSoft, Millipore). Data were analysed with R (v.4.0.3)^54^ and the packages CytoExploreR (v.1.0.8)^56^ and ggcyto (v.1.18.0)^57^ Cells with typical FSC and SSC parameters were selected and the median fluorescence intensity calculated for each sample. Each condition was measured in triplicate.

### Synthesis and characterization of TAMRA-D-R_8_

TAMRA-D-R_8_ was synthesized via manual Fmoc solid-phase peptide synthesis, using Fmoc-Rink amide resin (loading, 0.19 mmol g^−1^), as previously described^51^. TAMRA-D-R_8_ was obtained after RP-HPLC purification with an overall yield of 17% (15 mg) in 99% purity. It was characterized on an RP-HPLC Agilent SB-C18 column, H_2_O (0.1% TFA)/CH_3_CN (0.1% TFA) 95:5→5:95 (0→12 min)]. *R*_t_, 5.96 min. MS (ESI): 1,124.7 (9, [M+2H+4TFA]^2+^), 1,067.9 (17, [M+2H+3TFA]^2+^), 1,011.0 (14, [M+2H+2TFA]^2+^), 712.4 (37, [M+3H+3TFA]^3+^), 674.2 (100, [M+3H+2TFA]^3+^), 636.3 (95, [M+3H+TFA]^3+^), 598.2 (36, [M+3H]^3+^), 534.5 (24, [M+4H+3TFA]^4^^+^), 506.0 (36, [M+4H+2TFA]^4+^), 477.5 (48, [M+4H+TFA]^4+^), 449.1 (62, [M+4H]^4+^).

### Reporting summary

Further information on research design is available in the Nature Research Reporting Summary linked to this paper.

## Online content

Any methods, additional references, Nature Research reporting summaries, source data, extended data, supplementary information, acknowledgements, peer review information; details of author contributions and competing interests; and statements of data and code availability are available at 10.1038/s41586-022-04413-w.

### Supplementary information

Supplementary Information Supplementary Notes 1–3 and Figs. 1–3.

Reporting Summary

### Source data

Source Data Fig. 2

Source Data Extended Data Fig. 1

Source Data Extended Data Fig. 2

Source Data Extended Data Fig. 3

Source Data Extended Data Fig. 4

Source Data Extended Data Fig. 5

Source Data Extended Data Fig. 6

Source Data Extended Data Fig. 7

Source Data Extended Data Fig. 8

## Figures and Tables

**Extended Data Table 1:**
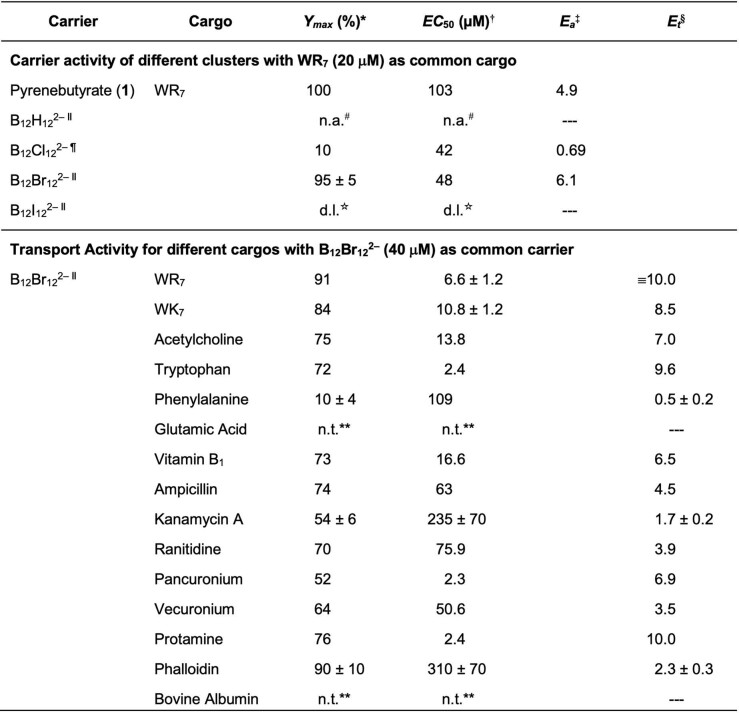
Characteristic membrane transport parameters of boron clusters for selected analytes (cargos) in vesicles determined by Hill analysis

* Maximal activity; 10% error (*SD*), unless stated differently. ^**†**^ Effective concentration to reach 50% activity of *Y*_max_; 10% error (*SD*), unless stated differently. ^**‡**^ Activator efficiency; 10% error (*SD*, calculated by considering error propagation with respect to both, *Y*_max_ and *EC*_50_). ^§^ Transport efficiency; 5% error (*SD*, calculated by considering error propagation with respect to both, *Y*_max_ and *EC*_50_), unless stated differently. ^ǁ^ Used as sodium salt. ^¶^ Used as caesium salt. ^♯^ n.a. = no detectable activity. ^☆^ d.l. = dye leakage, no determination possible. ** n.t. = no detectable transport.

## Data Availability

All data, including Source Data, are available with the paper. Source data are provided with this paper.

## Code availability

Data processing and fitting results can be generated by using numerical methods described in the Methods.
