# Supplementary material for: Boron clusters as broadband membrane carriers
Source: Nature. 2022 Mar 23;603(7902):637–42. doi: 10.1038/s41586-022-04413-w (PMC8942850; doi:10.1038/s41586-022-04413-w)
Supplement: Supplementary file 1 — Supplementary Notes 1–3 and Figs. 1–3. [file 41586_2022_4413_MOESM1_ESM.pdf]

nature portfolio

https://doi.org/10.1038/s41586-022-04413-w

Supplementary information

Boron clusters as broadband membrane carriers

In the format provided by the authors and unedited

Supplementary Notes and Figures

Supplementary Note 1: Time course experiments of TAMRA-R_8 delivery

Control experiments for TAMRA-R_8 delivery by using boron clusters with higher ($B_{12}Br_{12}^{2-}$) and lower ($B_{12}H_{12}^{2-}$) chaotropicity, imaged after different incubation times (10 min to 2 h), showed comparable results (Supplementary Fig. 1). These experiments support the conclusion that the difference in the observed TAMRA-R_8 delivery is due to the chaotropic character and cluster type, and confirms that the hydrogenated parent cluster does not lead to cytosolic peptide transport even at the longest selected incubation time.

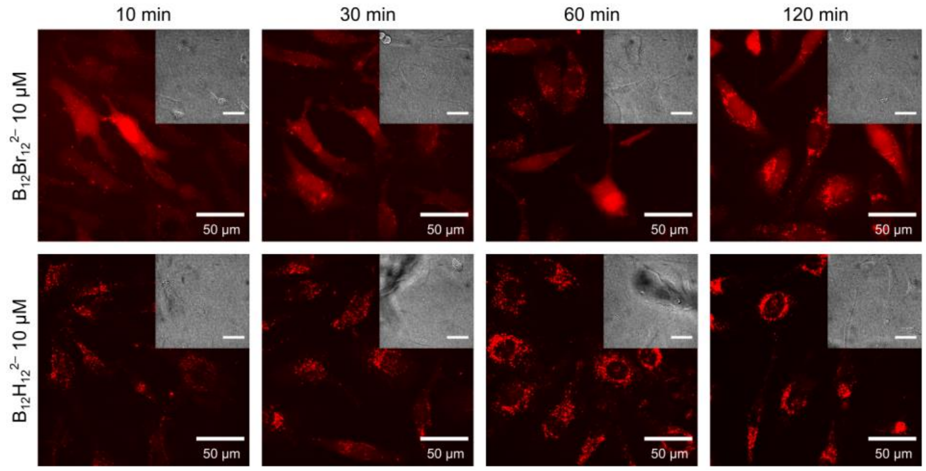

Supplementary Fig. 1 | Time course of TAMRA-R_8 uptake mediated by boron clusters of varying chaotropicity. HeLa cells were incubated with 1 µM TAMRA-R_8 in the presence of 10 µM $B_{12}Br_{12}^{2-}$ (upper row) or $B_{12}H_{12}^{2-}$ (lower row) diluted in HKR buffer for 1 h, washed with 0.1 mg/mL heparin in HKR, and imaged in DMEM w/o phenol red by confocal fluorescence microscopy after 10, 30, 60, and 120 min (left to right). Images show TAMRA-R_8 fluorescence (red) and the brightfield in the insets; scale bars: 50 µm.

Supplementary Note 2: Sequence of addition of cluster and phalloidin

According to the dynamic nature of chaotropic interactions, transport should be insensitive to the sequence of addition of cluster and cargo. To demonstrate this, cellular uptake of phalloidin-TRITC after varying sequences of addition and pre-incubation was investigated (Supplementary Fig. 2). Regardless of whether cluster was added first and phalloidin later, or the reverse, or whether both were added at the same time, with or without a pre-incubation time, efficient phalloidin transport and actin staining was observed in all cases. This robustness can be an advantage in comparison to methods that require a previous adsorption or fixation of the carrier to the cargo. In alternative approaches that require adsorption or encapsulation, e.g., lipofection, the order of mixing and the incubation time between cargo and carrier affects the transport efficiency and, thus, it needs to be carefully optimized for a successful delivery. The actual insensitivity of the boron clusters to the incubation process, in vesicles and in cells, rules out the possibility of any pre-adsorption requirement.

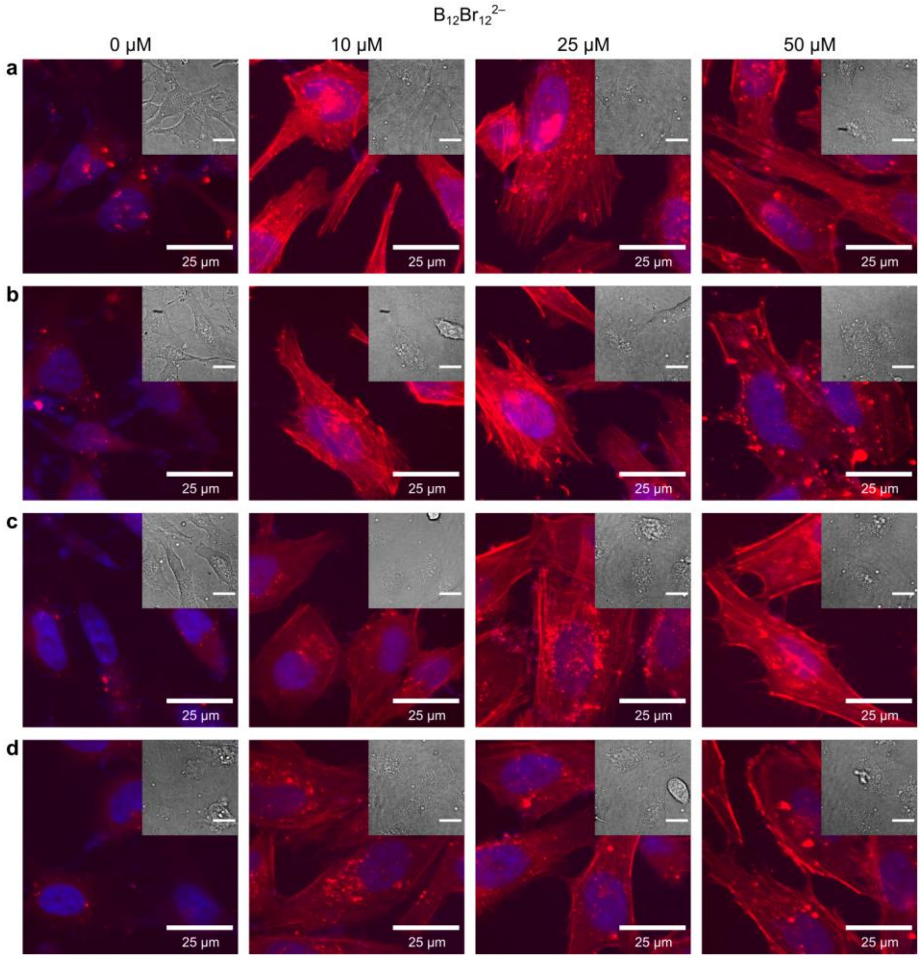

Supplementary Fig. 2 | $B_{12}Br_{12}^{2-}$-assisted phalloidin-TRITC transport into living cells with different sequence of addition and incubation modes. HeLa cells were treated with 2.5 µM phalloidin-TRITC (red) in the absence and presence of different concentrations of $B_{12}Br_{12}^{2-}$ cluster (10, 25, and 50 µM; left to right column) in HKR buffer for 3 h with varying sequence of addition: **a**, by adding first phalloidin-TRITC (cargo) to the cells, subsequently the cluster; **b**, by adding first the cluster, subsequently the cargo; **c**, by pre-mixing cargo and cluster and adding the mix to the cells immediately or **d**, same as in c, but after 20 min pre-incubation of the cargo-cluster mixture. In the next step, cells were stained with Hoechst (blue), washed for 5 min with 0.1 mg/mL Heparin and HKR buffer, and imaged by confocal fluorescence microscopy; brightfield images in insets, inset scale bars: 50 µm.

Supplementary Note 3: Non-conjugated TAMRA is not transported by the cluster

To rule out a potential artefact in the TAMRA-R_8 peptide transport experiments, that is, fluorescence signals arising from degradation fragments or the TAMRA probe itself, several control experiments were performed. In addition to the control experiments using a hydrolysis-resistant peptide (the enantiomeric peptide TAMRA-D-R_8, see main text and Extended Data Fig. 6) and the HPLC analysis of cytosolic extracts (Extended Data Fig. 5d, e), we performed transport experiments with the non-conjugated TAMRA probe (Supplementary Fig. 3). These experiments revealed that – in contrast to the labelled TAMRA-R_8 peptide – the isolated fluorescent dye is not carried by $B_{12}Br_{12}^{2-}$. Even at fifty times higher dye concentrations than the peptide, no relevant TAMRA fluorescence signal was observed in the cells.

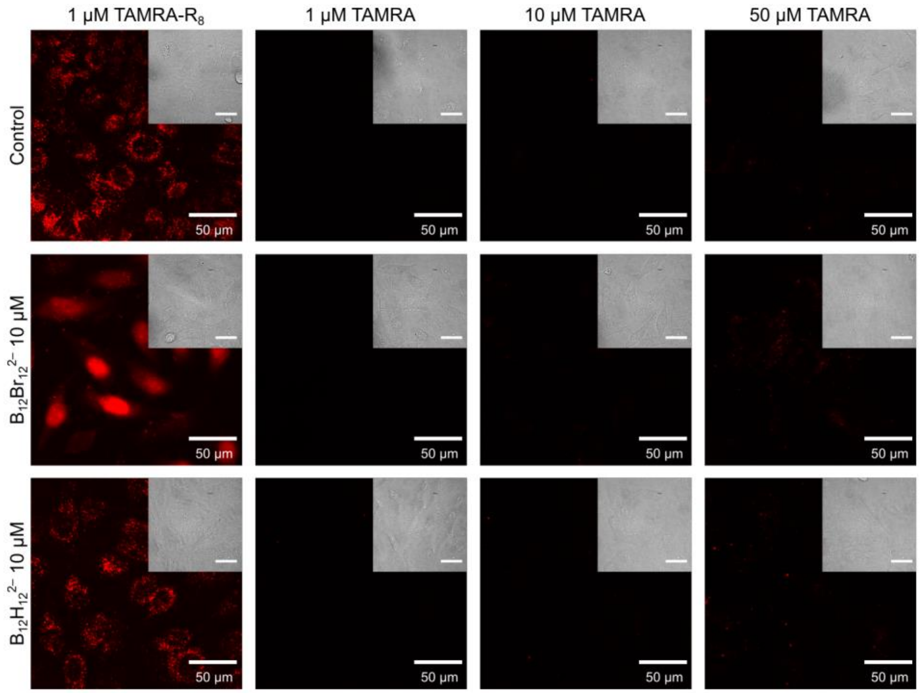

Supplementary Fig. 3 | TAMRA uptake control experiments in HeLa cells. Cells were incubated with 1 µM TAMRA-R_8 (as a positive control, left) and 1, 10, or 50 µM non-conjugated TAMRA (second to fourth columns) in the absence (top row) or in the presence of either 10 µM $B_{12}Br_{12}^{2-}$ or 10 µM $B_{12}H_{12}^{2-}$ (middle and bottom) diluted in HKR buffer for 1 h, washed for 5 min with 0.1 mg/mL heparin and HKR buffer, and imaged by confocal fluorescence microscopy. Where detectable, images show TAMRA-R_8 or TAMRA fluorescence (red) and the brightfield in the insets; scale bars: 50 µm.
