# Supplementary material for: Boron clusters as broadband membrane carriers
Source: Nature. 2022 Mar 23;603(7902):637–42. doi: 10.1038/s41586-022-04413-w (PMC8942850; doi:10.1038/s41586-022-04413-w)
Supplement: Supplementary file 2 — Reporting Summary [file 41586_2022_4413_MOESM2_ESM.pdf]

nature research

Corresponding author(s): Javier Montenegro, Werner M. Nau

Last updated by author(s): 2021/11/21

Reporting Summary

Nature Research wishes to improve the reproducibility of the work that we publish. This form provides structure for consistency and transparency in reporting. For further information on Nature Research policies, see our Editorial Policies and the Editorial Policy Checklist.

Statistics

For all statistical analyses, confirm that the following items are present in the figure legend, table legend, main text, or Methods section.

n/a	Confirmed	
☐	☒	The exact sample size (*n*) for each experimental group/condition, given as a discrete number and unit of measurement
☐	☒	A statement on whether measurements were taken from distinct samples or whether the same sample was measured repeatedly
☒	☐	The statistical test(s) used AND whether they are one- or two-sided<br>*Only common tests should be described solely by name; describe more complex techniques in the Methods section.*
☒	☐	A description of all covariates tested
☒	☐	A description of any assumptions or corrections, such as tests of normality and adjustment for multiple comparisons
☐	☒	A full description of the statistical parameters including central tendency (e.g. means) or other basic estimates (e.g. regression coefficient) AND variation (e.g. standard deviation) or associated estimates of uncertainty (e.g. confidence intervals)
☒	☐	For null hypothesis testing, the test statistic (e.g. *F*, *t*, *r*) with confidence intervals, effect sizes, degrees of freedom and *P* value noted<br>*Give P values as exact values whenever suitable.*
☒	☐	For Bayesian analysis, information on the choice of priors and Markov chain Monte Carlo settings
☒	☐	For hierarchical and complex designs, identification of the appropriate level for tests and full reporting of outcomes
☒	☐	Estimates of effect sizes (e.g. Cohen's *d*, Pearson's *r*), indicating how they were calculated

Our web collection on statistics for biologists contains articles on many of the points above.

Software and code

Policy information about availability of computer code

Data collection	Fluorescence measurements were recorded with a Jasco FP-8500 spectofluorometer equipped with temperature controller. Dynamic light scattering experiments were carried out on a Malvern Instruments DTS Nano 2000 Zeta-Sizer. Confocal images were acquired with a Dragonfly spinning disk confocal module (Andor) mounted on a Nikon Eclipse Ti-E equipped with an Andor Zyla 4.2 PLUS sCMOS camera, using Fusion software (Andor). Absorbance measurements were done in a Tecan Infinite F200Pro reader. Flow cytometry data were acquired with a Guava easyCyte BG HT flow cytometer using the software InCyte v. 3.2 (GuavaSoft, Millipore). For ICP-MS, an Agilent 7700x instrument was used.
Data analysis	Images were processed with FIJI (v. 2.1.0/1.53e). Data analysis and plotting were done with R (4.0.3), or Origin (v. 7.0 & v. 8.0). Flow cytometry data were analyzed on R (v. 4.0.3) using ggCyto (v. 1.18.0) and CytoExploreR (v. 1.0.8). Source data are deposited as Microsoft Excel files (v. 16.16.27).

For manuscripts utilizing custom algorithms or software that are central to the research but not yet described in published literature, software must be made available to editors and reviewers. We strongly encourage code deposition in a community repository (e.g. GitHub). See the Nature Research guidelines for submitting code & software for further information.

Data

Policy information about availability of data

All manuscripts must include a data availability statement. This statement should provide the following information, where applicable:

- Accession codes, unique identifiers, or web links for publicly available datasets
- A list of figures that have associated raw data
- A description of any restrictions on data availability

All data, including Source Data, are available with the paper.

Field-specific reporting

Please select the one below that is the best fit for your research. If you are not sure, read the appropriate sections before making your selection.

☒ Life sciences ☐ Behavioural & social sciences ☐ Ecological, evolutionary & environmental sciences

For a reference copy of the document with all sections, see nature.com/documents/nr-reporting-summary-flat.pdf

Life sciences study design

All studies must disclose on these points even when the disclosure is negative.

Sample size	No statistical methods were used to determine sample size. Sample sizes were chosen based on our previous experience with similar experiments and previous studies on delivery of cargos into cells (10.1039/c7sc03918b, 10.1038/s41467-020-17997-6, 10.1016/j.chembiol.2012.05.022, 10.1038/nchem.2779, 10.1128/AAC.01786-10).
Data exclusions	No data were excluded from the analysis.
Replication	All experiments were performed at least twice. All attempts of replication were successful.
Randomization	There were no variables or interventions to randomize in this study. In vitro cell experiments were performed under controlled conditions, and all experiments were carried out with their corresponding controls in parallel to minimise experimental variation.
Blinding	Blinding was not performed, as the same person carried out the experiments and the analysis of the results. Several experiments were repeated by another researcher to confirm the results.

Reporting for specific materials, systems and methods

We require information from authors about some types of materials, experimental systems and methods used in many studies. Here, indicate whether each material, system or method listed is relevant to your study. If you are not sure if a list item applies to your research, read the appropriate section before selecting a response.

Materials & experimental systems

n/a	Involved in the study	
☒	☐	Antibodies
☐	☒	Eukaryotic cell lines
☒	☐	Palaeontology and archaeology
☒	☐	Animals and other organisms
☒	☐	Human research participants
☒	☐	Clinical data
☒	☐	Dual use research of concern

Methods

n/a	Involved in the study	
☒	☐	ChIP-seq
☐	☒	Flow cytometry
☒	☐	MRI-based neuroimaging

Eukaryotic cell lines

Policy information about cell lines

Cell line source(s)	HeLa, HEK293,ARPE-19, A549: ATCC<br>GT1-7, GnRH: Millipore
Authentication	None of the cell lines were authenticated.
Mycoplasma contamination	Cells were not tested for mycoplasma contamination.
Commonly misidentified lines<br>(See ICLAC register)	None of the cells used are listed in the ICLAC database.

April 2020

Flow Cytometry

Plots

Confirm that:

☒ The axis labels state the marker and fluorochrome used (e.g. CD4-FITC).

☒ The axis scales are clearly visible. Include numbers along axes only for bottom left plot of group (a 'group' is an analysis of identical markers).

☒ All plots are contour plots with outliers or pseudocolor plots.

☒ A numerical value for number of cells or percentage (with statistics) is provided.

Methodology

Sample preparation	HeLa cells were seeded on 96-well plates at 10,000 cells/w. The next day, they were incubated for 1 h with the indicated compounds diluted in HKR. Cells were washed for 5 min with HKR containing 0.1 mg/mL heparin, washed again with HKR, and trypsinized. Trypsin was neutralized with PBS containing 2% FBS and 5 mM EDTA.
Instrument	easyCyte BG HT Flow cytometer (Millipore)
Software	Acquisition: InCyte from GuavaSoft 3.2 (Millipore).<br>Analysis: R (v. 4.0.3) with packages CytoExploreR (v. 1.0.8) and ggcyto (v. 1.18.0).
Cell population abundance	Cells were not sorted. 2500 events were acquired for each well.
Gating strategy	Cells with typical FSC/SSC parameters were selected and the fluorescence of that population studied.

☒ Tick this box to confirm that a figure exemplifying the gating strategy is provided in the Supplementary Information.
